# Sexual orientation differences in treatment expectation, alliance, and outcome among patients at risk for suicide in a public psychiatric hospital

**DOI:** 10.1186/s12888-017-1337-8

**Published:** 2017-05-15

**Authors:** Martin Plöderl, Sabine Kunrath, Robert J. Cramer, Jen Wang, Larissa Hauer, Clemens Fartacek

**Affiliations:** 1 Department for Crisis Intervention and Suicide Prevention, Christian Doppler Clinic, Paracelsus Medical University, Ignaz Harrerstrasse 79, A-5020 Salzburg, Austria; 2 Department of Clinical Psychology, Christian Doppler Clinic, Paracelsus Medical University, Salzburg, Austria; 3 School of Community and Environmental Health Sciences, Old Dominion University, Norfolk, Virginia USA; 4 Virginia Consortium Program in Clinical Psychology, Norfolk, Virginia USA; 5 Interdisciplinary Division for Adolescent Health, Department of Pediatrics, Lausanne University Hospital, Lausanne, Switzerland

**Keywords:** Gay, Lesbian, Suicide, Treatment, Common factors

## Abstract

**Background:**

Sexual minority (SM) individuals (gay, lesbian, bisexual, or otherwise nonheterosexual) are at increased risk for mental disorders and suicide and adequate mental healthcare may be life-saving. However, SM patients experience barriers in mental healthcare that have been attributed to the lack of SM-specific competencies and heterosexist attitudes and behaviors on the part of mental health professionals. Such barriers could have a negative impact on common treatment factors such as treatment expectancy or therapeutic alliance, culminating in poorer treatment outcomes for SM versus heterosexual patients. Actual empirical data from general psychiatric settings is lacking, however. Thus, comparing the treatment outcome of heterosexual and SM patients at risk for suicide was the primary aim of this study. The secondary aim was to compare treatment expectation and working alliance as two common factors.

**Methods:**

We report on 633 patients from a suicide prevention inpatient department within a public psychiatric hospital. Most patients were at risk for suicide due to a recent suicide attempt or warning signs for suicide, usually in the context of a severe psychiatric disorder. At least one indicator of SM status was reported by 21% of patients. We assessed the treatment outcome by calculating the quantitative change in suicide ideation, hopelessness, and depression. We also ran related treatment responder analyses. Treatment expectation and working alliance were the assessed common factors.

**Results:**

Contrary to the primary hypothesis, SM and heterosexual patients were comparable in their improvement in suicide ideation, hopelessness, or depression, both quantitatively and in treatment responder analysis. Contrary to the secondary hypothesis, there were no significant sexual orientation differences in treatment expectation and working alliance. When adjusting for sociodemographics, diagnosis, and length of stay, some sexual orientation differences became significant, indicating that SM patients have better outcomes.

**Conclusions:**

These unexpected but positive findings may be due to common factors of therapy compensating for SM-specific competencies. It may also be due to actual presence of SM competencies – though unmeasured – in the department. Replication in other treatment settings and assessment of SM-specific competencies are needed, especially in the field of suicide prevention, before these findings can be generalized.

**Electronic supplementary material:**

The online version of this article (doi:10.1186/s12888-017-1337-8) contains supplementary material, which is available to authorized users.

## Background

Compared to heterosexuals, sexual minority (SM) individuals are at increased risk for mental disorders, suicide attempts, and suicide [1–3]. SM is an umbrella term that covers different populations of individuals who are not exclusively heterosexual. SM include individuals with nonheterosexual identity (gay, lesbian, bisexual, mostly heterosexual, queer, questioning, etc.), nonexclusive heterosexual behavior (bisexual or same-sex behavior), or nonexclusive heterosexual attraction. These subpopulations share an increased risk for mental health disorders and suicide, across gender, country, or year of study [2]. Sexual orientation disparities are large for suicides and suicide attempts but also notable for disorders known to be associated with increased suicide risk, especially depression and substance - related disorders [4, 5]. Data on other Axis I and Axis II disorders are sparse but generally suggest an increased risk for SM individuals, too [2]. Explanatory models about this increased risk center around general stressors and stigma-related stressors that are specific for sexual minorities: For example, such stressors include experiences and fear of discrimination and violence because of one’s sexual orientation, internalized homophobia, stress associated with hiding one’s sexual orientation, lack of social support [6–9]. The findings of recent studies support these explanatory models (e.g., [10–15]).

### Do SM individuals benefit less from mental healthcare treatment?

SM individuals have been recognized as a target group for mental health intervention [16] and suicide prevention [3]. However, SM patients seem to face interpersonal and structural barriers to appropriate adequate healthcare [16] that resemble enacted and expected stigma [17] within healthcare systems. On an interpersonal level, there may be experiences of discrimination, microaggressions, harsh language, rejection, denial of service, attempts to change sexual orientation, silencing sexual orientation issues, or implicit biases [16, 18–24]. On an intrapersonal level, there may be expectations of these behaviors, hindering disclosure to health professionals [16]. On a structural level, healthcare providers often lack cultural competency with respect to SM and gender minority issues and experience difficulties in providing SM-affirmative healthcare [16, 24–28]. For example, 85% of medical students reported lacking SM-specific healthcare education across all years in a recent UK study [26]. Given these barriers, it is not surprising that SM patients report lower levels of satisfaction with mental healthcare than heterosexuals [29], and remain frequently undisclosed or avoid sexual orientation issues altogether [19, 30–32]. Consequently, there is interest in and demand for SM-specific treatment [3, 16, 33]. Despite a growing number of institutions specializing in healthcare for SM individuals, specific interventions remain exceptions in most settings, meaning that most SM individuals will receive usual care.

Based on the personal and structural barriers described above, it can be hypothesized that SM patients may benefit less from usual mental healthcare. This is especially problematic in the case of suicidal SM patients, where appropriate treatment could be life-saving. Knowing whether SM individuals actually derive less benefit from general psychiatric treatment is crucial to understand the scope of the problem and the urgency for necessary changes in mental healthcare. Thus, the primary goal of our study is to explore whether SM and heterosexual patients at risk for suicide differ in their benefit from treatment in a setting that is not specifically tailored for SM patients.

To our knowledge, no studies have been published which assess how SM patients benefit from regular treatment in psychiatric care, including psychiatric suicide prevention measures such as inpatient crisis intervention. This gap is likely a result of the lack of systematic assessment of sexual orientation in healthcare settings. Most studies used selected samples of sexual minority individuals who reported their healthcare experiences retrospectively (e.g., [20, 22, 32, 34, 35]), and it is not possible to assess the actual impact of barriers on outcomes among SM patients from these samples. In our study, we collected information on sexual orientation from all patients in a treatment setting. Thus, selection for treatment and selection for study participation did not vary with the sexual orientation of patients, facilitating assessment of potential differences in treatment effects between SM and heterosexual patients.

### Are common treatment factors less optimal for SM patients?

Beyond identifying the mere difference in treatment outcome for SM versus heterosexual patients, a secondary goal of our paper is to explore the impact of mechanisms known to be important for successful treatment by sexual orientation. In the contextual model of psychotherapy [36], contextual factors for treatment success have been outlined: empathic and real therapeutic relationship, creation of positive expectations, shared explanations of the problem, agreement on treatment goals, tasks, and therapeutic and healthy actions. Research has shown that a good therapeutic alliance is crucial in suicide prevention as well [37–40]. Furthermore, a positive treatment expectation and a good patient-clinician relationship are important beyond psychotherapeutic treatments, for example in pharmacotherapy, where the two factors enhance effectiveness of and adherence to the treatment of psychiatric disorders [36, 41, 42].

Given the interpersonal and structural barriers that SM patients have reported, it is plausible that common factors such as treatment expectancy and therapeutic relationship may be impaired. Some patients may fear rejection or even discrimination from mental health professionals, leading to a negative expectation that treatment would be helpful. For older patients, this expectation may stem from the long-standing pathologization of SM individuals in psychiatry. Impaired treatment expectations may also result from previous negative experience in healthcare, or via projections of negative societal experiences onto therapeutic settings. Therapeutic alliance may be influenced negatively by impaired treatment expectancy, by the lack of cultural competency of healthcare professionals, or even by verbal or nonverbal discrimination.

Based on the theoretical assumption outlined above, our primary hypothesis is that treatment outcome is less optimal for SM compared to heterosexual patients. According to the secondary hypothesis, treatment expectation and working alliance as two common factors of treatment are similarily less optimal for SM than heterosexual patients.

## Methods

### Participants and procedure

Between the assessment period of February 5, 2010, to February 26, 2014, 997 admissions of individual patients were recorded at the department of crisis intervention and suicide prevention (CI-SP), which is part of the public psychiatric hospital of the City of Salzburg, Austria (“Christian Doppler Clinic”). The province of Salzburg is the sole proprietor of the hospital. There is no other psychiatric hospital in the region; thus, nearly all patients who require inpatient treatment are admitted to this hospital. The CI-SP department belongs to one of five departments for patients with acute mental disorders and is specialized in suicide prevention. Adult patients (18 years or older) are selected for treatment in CI-SP department predominantly after suicide attempts or other crisis situations with warning signs of suicide, such as suicide ideation, despair, or hopelessness [43]. Usually, patients’ suicidal crisis occurs within the context of a psychiatric disorder, most often affective disorders with a current depressive episode, adjustment disorders, and frequently comorbid with substance - related disorders or Cluster B Axis II personality disorders. There is consensus that such patients are at heightened risk for suicide [44]. Patients with acute psychotic disorders are usually not treated since the treatment-method is talk-oriented crisis intervention (see below). Infrequently, other patients are treated at the CI-SP department, since there is a mandatory admission for all patients in Salzburg with acute mental health disorders and sometimes open beds are unavailable at other departments.

The treatment method is crisis intervention, with a planned stay of 3–14 days and with longer stays in cases of enduring major depressive episodes or other factors complicating discharge (e.g., lack of housing), or temporary transfers to the closed ward in case of imminent suicide risk. Following usual crisis intervention plans, high-frequency meetings between patient and his/her responsible psychotherapist or clinical psychologist form the modality of treatment. Crisis intervention steps are assessing suicide risk, establishing rapport, analyzing the problem in a collaborative way, managing feelings, exploring alternatives, developing a positive plan, safety planning, and follow-up [45]. Crisis intervention is adapted for patients with borderline personality disorder by including elements of Dialectical Behavior Therapy [46]. Additional forms of treatment are applied to support and realize crisis intervention and include medication, psychiatric nursing, occupational therapy, physiotherapy, relaxation groups, and psychoeducational groups. Since most of the patients suffer from acute mental disorders, most frequently a depressive episode, nearly all of the patients receive antidepressants. Common additional medications are antipsychotics, mood-stabilizers, and benzodiazepines. The medication is adapted in daily meetings with a psychiatrist.

At admission, the responsible psychiatrist and psychotherapist/psychologist interview the patient in a narrative style to enhance therapeutic alliance. This is followed by a structured assessment of psychiatric diagnosis, physical symptoms, suicidality (suicide ideation, plans, self-control), previous suicide attempts (date, reason, method, medical treatment), nonsuicidal self-injury, aggressive behavior, and other treatment-relevant information. A realistic short-term goal for crisis intervention is agreed upon (e.g., enhancing sleep, managing depressive symptoms, reducing suicide ideation). Additional treatments mentioned above are prescribed individually. The multiprofessional team meets daily from Monday to Friday to discuss suicide risk, evaluate and modify crisis intervention steps or goals, manage transference and countertransference issues, evaluate and modify the diagnosis, and plan for discharge.

Exclusion criteria for the present study were lack of language skills because of migration from non-German speaking countries, discharge or transfer to another unit before completing the assessment, dementia, and acute psychotic symptoms. Two assessments were scheduled. The intake assessment was completed after the intake interview with the responsible psychiatrist and psychologist/psychotherapist. For this assessment, eligible patients filled out a set of questionnaires online, if possible within the first two days of their stay. The questionnaires assessed suicide-related risk- and protective factors, sociodemographic variables, and treatment expectation (see below for the instruments used for this study and [47] for other instruments not used in this study). In case of acute psychosis or severe depression, patients completed the questionnaires as soon as their condition improved. The second assessment was scheduled within two days before discharge and included only a subset of questionnaires on suicide ideation, depression, hopelessness, and working alliance. If the length of stay was too short, no additional assessment was made before discharge.

Patients were informed that data were stored safely (on a separate server only accessed by selected researchers and without identifying names except the protected clinic code) and that the data could only be used by the research team and the responsible clinician. The responsible clinicians were only informed about certain outcome variables (suicide ideation, hopelessness, depression, treatment expectancy, working alliance) but not about sexual orientation variables. All study participants gave written consent. The study was approved by the ethics commission of Salzburg.

### Instruments

#### Sexual orientation

Following recommendations [48], the intake assessment included three dimensions of sexual orientation. Sexual attraction was assessed with the item “To whom are you sexually attracted?” (men, women, men and women), with the possible response options in brackets. Responses were categorized in heterosexual, bisexual, and same-sex attraction, and the latter categories were subsumed under the SM attraction category.

Sexual behavior was solicited with two items: “With how many men did you ever have sex with?” (with none, one man, two men, three or more men) and a corresponding item for sex with women. Responses were categorized into heterosexual (exclusively other-sex sexual behavior), bisexual (sex with both men and women), same-sex (exclusively same-sex behavior), and a separate “no sex” category. Homosexually or bisexually active participants were collapsed into the SM behavior category.

Sexual identity was assessed with “What describes your sexual orientation best?” [heterosexual (sexually interested in the other sex), predominantly heterosexual, bisexual (sexually interested in men as well as women), gay/lesbian/homosexual (sexually interested in the same sex), I am not sure, I don’t understand this question, other]. In contrast to the items on behavior and attraction, participants also had an open response field after the “other” option. We decided to include “homosexual” as a response option, since in Austria, some still prefer this term to “schwul” (the German expression for “gay”).

Since subgroups of sexual orientation were too small to allow statistical analysis with adequate power, we created a binary sexual orientation variable. Patients were classified as SM if they had any indicator of SM in the identity, behavior, or attraction variable. All others were classified as heterosexual. This binary variable was used for all analysis. Results for each of the three dimensions of sexual orientation may be obtained upon request.

#### Treatment outcome

The three relevant variables were assessed at intake and before discharge. Suicide ideation was measured with Beck’s Scale for Suicide Ideation [49] (Cronbach’s alpha *r*_α_ = .94 for Items 1 to 19); suicide attempt status (yes/no) was derived from the related item of that scale. Hopelessness was assessed with Beck’s Hopelessness-Scale [50] (*r*_α_ = .88), depression with Beck’s Depression Inventory, Version II [51] (*r*_α_ = .91). These three instruments are typically used to assess treatment outcome in related studies [52–55], since they are established measures of suicide risk [56, 57] with sound psychometric properties [57, 58] and therefore recommended in suicide prevention research [59]. The calculation of the outcome measures (intake-discharge quantitative differences and treatment responder analysis) is described below.

#### Treatment expectancy and working alliance

Treatment expectancy was included in the intake assessment with the item “How much do you trust that the treatment at this department will help you solve your problems?” Participants had to respond on a visual-analogue scale ranging from 0 “no trust” to 100 “maximum trust.” A similar procedure had been used previously [60]. Therapeutic alliance was included in the discharge assessment, using the German translation of the Working Alliance Inventory [61] (*r*_α_ = .94). Examples of items are “I believe my therapist likes me,” or “My therapist and I collaborate on setting goals for my therapy.” Four patients had more than two missing items in this questionnaire; following the manual [61], we did not calculate a score for these patients. We replaced one or two missing value(s) with the median of the completed items in *n* = 19 patients.

#### Psychiatric diagnosis

Patients were first diagnosed according to the ICD-10 in the initial intake interview with a psychiatrist and a psychologist or psychotherapist and by studying the medical records or the diagnosis given by referring institutions. Each patient then had daily contact with a psychiatrist, a clinical psychologist or psychotherapist, and a nurse. In the daily meetings of the multiprofessional team, the appropriateness of the diagnosis was discussed and, if necessary, adjusted. The diagnosis at discharge was used as a control variable for the present study. Only the main categories of the ICD-10 (F0 – F9) were used for the analysis (see Table 1).

**Table 1:** Distribution of sexual orientation

Indicator of Sexual Orientation	*n*	(%)
Any indicator of SM		
Heterosexual	502	(79)
SM	131	(21)
Sexual Behavior		
Heterosexual	528	(83)
Bisexual	68	(11)
Same-Sex	6	(1)
No Sexual Contacts	31	(5)
Sexual Attraction		
Heterosexual	572	(90)
Bisexual	31	(5)
Same-Sex	30	(5)
Sexual Identity		
Heterosexual	489	(77)
Predominantly heterosexual	42	(7)
Bisexual	14	(2)
Gay/Lesbian	16	(3)
Not sure	16	(3)
Do not understand question	41	(6)
Other	15	(2)
Sexual Identity - Clarified^a^		
Heterosexual	542	(86)
Predominantly heterosexual	42	(7)
Bisexual	14	(2)
Gay/Lesbian	16	(3)
Not sure	16	(3)
Other nonheterosexual	3	(0)

Note:

^a^After categorizing patients who responded with “other” on the identity item, based on their qualitative responses and on their responses to the sexual behavior and sexual attraction items (see Results section)

#### Sociodemographics

Single items were used to assess gender, income, nationality, and mother language. Age was calculated from the date of birth. Degree of education was coded ordinally as compulsory schooling (1), compulsory schooling plus apprenticeship (2), A-level (3), and academic degree (applied sciences: 4, regular university: 5). Since 72 participants replied with “other,” this could not be categorized ordinally. They were coded as missing and then imputed with R’s “transcan” function [62] by using all other sociodemographic variables for prediction. Similarly, since the item on income was not mandatory, missing data (*n* = 91, 14%) were imputed.

#### Length of stay

This was calculated from the date of admission and discharge from the CI-SP department. In some cases, this also included days of temporary transfers to other departments, most often the closed ward. Since length of stay was skewed (a few patients had very long stays, range 3–422), we log-transformed the variable in the multivariate analysis.

### Statistical analysis

We used two measures for treatment outcome. First, quantitative change was measured by calculating the differences between intake and discharge levels of suicide ideation, hopelessness, and depression. Sexual orientation differences (SM vs. heterosexual patients) were then calculated with *t*-tests and with multivariate linear regression models to adjust for potential confounders. Second, we used a treatment responder analysis [63]. To be classified as a responder, the intake level had to be above the clinical cut-off with an improvement of at least 50% of the clinical range. Furthermore, the overall improvement had to be at least 25% in the general range of the scale. This is important because if a patient’s initial level is only slightly above the cut-off, then it is too easy to gain >50% possible change in the clinical range. Nonresponders were those who did not achieve the improvement just described. Those who were not in the pathological range in the intake assessment were excluded from the responder analysis. Sexual-orientation differences in responder status were based on simple odds ratios or on multivariate logistic regression models to adjust for potential confounders.

Potential confounding variables in this study were sociodemographics (gender, age, level of education, income, nationality, mother language), length of stay, and diagnosis (F0-F9 codes). Since there are 18 variables, we first ran stepwise regression analysis with backwards elimination with only the sociodemographics and with only the 10 diagnoses to identify important predictors for the dependent variable in question. We also inspected interactions of each confounding variable with sexual orientation (detailed results in supplement). Significant interactions are reported and also included in the full model. Regression results for the full models are reported in detail in Additional file 1.

For a priori power analysis, we assumed a prevalence of 15% SM individuals in our sample. To detect small effects (*d* = 0.3) with *ɑ* = .05 and 80% power for comparing means of two independent samples (SM vs. heterosexuals) with one-sided *t*-tests, a sample size of 529 heterosexuals and 79 SM individuals would be needed (671 and 101 for two - tailed tests, respectively). Our sample included 502 heterosexuals and 131 SM patients, thus a post hoc analysis revealed an actual power of 92% in one-sided and 86% in two-sided *t*-tests [64].

Missing data were impossible for most variables, since each item had to be completed in order to move on to the next item. Due to a programming error, however, it was possible to skip items or to fill in impossible values only for a few variables at the very beginning of the assessment period and only for a few variables as this was corrected early. Details on imputation are given above for the corresponding instruments.

We calculated Cohen’s *d* as a measure of effect for continuous variables size by dividing the differences between the measurements of women and men by the pooled standard deviation [65]. For categorical data, odds ratios (*OR*) were used to quantify effect sizes [66, 67]. To determine the associations between variables we used Spearman rank correlation. We used SPSS Version 21 [68] and R 3.1.3 [69] for statistical analysis.

## Results

### Sample description

Out of a total of 997 patients admitted during the assessment period, 834 (84%) fulfilled the inclusion criteria for the intake assessment, and 633 (63%) completed both intake and discharge assessments, comprising the analyzed sample.

With respect to sexual orientation, 12% reported bisexual or same-sex behavior and 10% reported same-sex or bisexual attraction (Table 1). For the identity item, nearly all patients who chose the “I don’t understand” or “other” response either reported exclusive heterosexual behavior/attraction or sometimes reported a related qualitative statement (e.g., “I am normal”). The few patients who responded with a SM identity (genderqueer, open to anybody, etc.) on the open-ended item also had SM behavior/attraction responses. After clarifying these “other” and “don’t understand” categories, 14% of patients were classified as having a SM identity. Overall, 21% reported at least one indicator of SM status.

### Differences by sexual orientation

#### Sociodemographic variables

SM patients were significantly younger (*d* = 0.36) and more likely female (*OR* = 1.59, 95%-*CI* 1.08-2.38), compared to heterosexual patients, but the differences were small. No other sexual orientation differences achieved statistical significance (Table 2).

**Table 2:** Sexual orientation differences in sociodemographics, diagnosis, and intake/discharge assessments of outcome variables

	Heterosexual *M* (*SD*) or *n* (%)	Sexual Minority *M* (*SD*) or *n* (%)	t.test or *OR* (95%-CI)	Cohen’s *d* (95%-*CI*)
Gender (% female)	257 (51)	82 (63)	1.59 (1.08–2.38)*	
Age	40.10 (12.57)	35.68 (12.20)	3.67 (207.89)**	0.36 (0.16–0.55)
Level of education^a^	2.43 (1.12)	2.35 (1.06)	0.83 (213.43)	0.08 (−0.11–0.27)
Income (Euro per month, net)	1271.94 (1118.94)	1130.81 (1298.88)	1.14 (183.44)	0.12 (−0.07–0.31)
Nationality (% Austrian)	442 (88)	116 (89)	0.96 (0.51–1.71)	
Mother language (% German)	452 (90)	119 (91)	0.92 (0.45–1.73)	
Length of stay	24.08 (24.61)	30.01 (34.00)	−1.87 (167.15)	−0.22 (−0.41 − −0.02)
Diagnosis				
F0: Mental disorders due to known physical conditions	20 (4)	2 (2)	0.40 (0.05–1.40)	
F1: Substance-related disorders	137 (27)	38 (29)	1.09 (0.69–1.66)	
F2: Psychotic disorders	25 (5)	6 (5)	0.93 (0.34–2.20)	
F3: Mood disorders	383 (76)	97 (74)	0.88 (0.57–1.39)	
F4: Anxiety, dissociative, stress-related, somatoform disorders	176 (35)	44 (34)	0.94 (0.62–1.40)	
F5: Disorders associated with physiological disturbances and physical factors	34 (7)	11 (8)	1.27 (0.60–2.52)	
F6: Personality disorders	90 (18)	38 (29)	1.87 (1.19–2.90)**	
F7: Mental retardation	26 (5)	5 (4)	0.74 (0.24–1.84)	
F8: Pervasive and specific developmental disorders	16 (3)	2 (2)	0.50 (0.07–1.81)	
F9: Disorders with onset in childhood/adolescence	6 (1)	4 (3)	2.63 (0.64–9.63)	
Intake Assessment				
Suicide Ideation	8.24 (8.64)	10.43 (9.00)	−2.49 (197.06)*	−0.25 (−0.44 − −0.06)
Hopelessness	30.78 (5.35)	31.36 (4.94)	−1.16 (216.43)	−0.11 (−0.30–0.08)
Depression	28.68 (12.07)	30.55 (11.34)	−1.66 (213.35)	−0.16 (−0.35–0.04)
Suicide attempt	224 (45)	70 (53)	1.42 (0.97–2.10)	
Discharge Assessment				
Suicide Ideation	3.44 (6.33)	5.12 (7.31)	−2.40 (184.01)*	−0.26 (−0.45 − −0.06
Hopelessness	27.24 (5.59)	27.69 (5.44)	−0.83 (207.62)	−0.08 (−0.27–0.11)
Depression	16.17 (11.68)	17.77 (12.57)	−1.32 (192.60)	−0.14 (−0.33–0.06)

Note:

*p < .05. **p < .01

^a^Level of education was coded ordinally as compulsory schooling (1), compulsory schooling plus apprenticeship (2), A-level (3), academic degree (applied sciences: 4, regular university: 5). 72 participants reported “other” education which could not be categorized ordinally and were thus coded as missing. Of note, a Chi-squared test using all education categories produced similar results

#### Length of stay

SM and heterosexual patients did not differ significantly in their length of stay (*d* = 0.22).

#### Diagnosis

The distribution of diagnosis only differed significantly for personality disorders, where SM patients were almost twice as likely to receive such a diagnosis, compared to heterosexual patients (Table 2).

#### Outcome variables

At both intake and discharge assessment, there were only small sexual orientation differences (*d* < 0.27) in suicide ideation, hopelessness, and depression. Only suicide ideation achieved statistical significance, indicating that SM patients had slightly higher levels than heterosexual patients at intake and discharge (Table 2).

### Treatment outcome (primary study aim)

#### Suicide ideation

##### Intake-discharge differences

There was a close to zero difference in the improvement of suicide ideation between heterosexual and SM patients in the unadjusted (*d* = 0.06) and adjusted analysis (Table 3). Only F7 diagnosis (mental retardation) interacted significantly with sexual orientation: SM patients with F7 diagnosis showed a large improvement in suicide ideation compared to heterosexual patients with F7 diagnosis (*d* = 1.19), whereas no sexual orientation difference occurred among patient without F7 diagnosis (*d* = 0.02).

**Table 3:** Sexual orientation differences in treatment outcome (primary hypothesis), working alliance and treatment expectation (secondary hypothesis)

	Heterosexual *M* (*SD*) or *n* (%)	Sexual Minority *M* (*SD*) or *n* (%)	*t*-test or *OR* (95%-*CI*)	Cohen’s *d* (95%-*CI*) Regression Coefficient (*SE*)
Treatment Outcome (Primary Hypothesis)				
Difference Intake vs. Discharge				
Suicide ideation	4.80 (7.76)	5.31 (7.88)	−0.66 (200.88)	−0.06 (−0.26–0.13)
				Unadjusted 0.51 (0.76)
				Adjusted^a^ 2.26 (3.65)
Hopelessness	3.55 (4.86)	3.67 (4.76)	−0.03 (206.18)	−0.03 (−0.22–0.17)
				Unadjusted 0.13 (0.479)
				Adjusted^b^ 0.82 (0.55)
Depression	12.51 (10.80)	12.78 (10.91)	−0.25 (201.46)	−0.02 (−0.22–0.17)
				Unadjusted 0.27 (1.06)
				Adjusted^c^ 11.78 (4.98)*
Responder Analysis				
Suicide Ideation				
Responder	194 (39)	57 (44)	Baseline	
Nonresponder	74 (15)	26 (20)	1.20 (0.69–2.04)	Adjusted^d^ 0.44 (0.18–1.05)
Nonpathological	234 (47)	48 (37)	-	
Hopelessness				
Responder	93 (19)	22 (17)	Baseline	
Nonresponder	290 (58)	86 (66)	1.25 (0.75–2.15)	Adjusted^e^ 2.01 (0.97–4.16)
Nonpathological	119 (24)	23 (18)	-	
Depression				
Responder	304 (61)	84 (64)	Baseline	
Nonresponder	127 (25)	37 (28)	1.06 (0.67–1.63)	Adjusted^f^ 0.77 (0.46–1.28)**
Nonpathological	71 (14)	10 (8)	-	
Secondary Hypothesis				
Treatment Expectancy (at intake)	73.13 (23.29)	73.70 (23.06)	−0.25 (204.65)	−0.02 (−0.22–0.17)
			Unadjusted	0.54 (2.28)
			Adjusted^g^	16.53 (8.06)*
Working Alliance (at discharge, *n* = 629)	47.54 (9.07)	48.37 (9.46)	−0.90 (193.23)	−0.09 (−0.28–0.10)
			Unadjusted	0.83 (0.90)
			Adjusted^h^	12.74 (5.06)*

Note:

^a^Adjusted for nationality, diagnosis (F3, F6, F7), length of stay, and interaction F7 x SM, and length of stay x SM

^b^Adjusted for income, mother language, diagnosis (F0, F3, F6, F8), length of stay, and interaction F6 x SM

^c^Adjusted for income, mother language, F3 diagnosis, length of stay, and interaction length of stay x SM

^d^Adjusted for gender, mother language, F6 diagnosis, length of stay, and the interactions F3 x SM, F6 x SM, and length of stay x SM

^e^Adjusted for nationality, length of stay, and F4 diagnosis (with interaction term F4 x SM)

^f^Adjusted for mother language, length of stay, and interaction of SM x length of stay

^g^Adjusted for age, education, income, diagnosis (F1, F3, F6, F8), length of stay, nationality, and nationality x SM

^h^Adjusted for age, income, mother language, F6 diagnosis, nationality, nationality x SM, length of stay, and length of stay x SM

*p < .05. **p < .01

##### Responder analysis

The odds for not responding to treatment were comparable between heterosexual and SM patients in the unadjusted analysis (*OR* = 1.20, 95%-*CI* 0.69–2.04). When adjusting for confounders, SM patients were somewhat less likely nonresponders, compared to heterosexuals (*OR* = 0.44, 95%-*CI* 0.18–1.05), but the difference did not reach statistical significance (*p* = .10). Significant interactions occurred for F3 diagnosis (affective disorders) and sexual orientation: Among patients without F3 diagnosis, SM patients were more likely nonresponders than heterosexual patients (*OR* = 3.56), whereas only a small sexual orientation difference was found among those with a F3 diagnosis (*OR* = 0.81). There was a reversed interaction effect for F6 diagnosis (personality disorders): Among patients without F6 diagnosis, SM patients were less likely nonresponders than heterosexual patients (*OR* = 0.64), whereas the reverse association was found among those with a F6 diagnosis (*OR* = 2.65). Finally, length of stay interacted with sexual orientation: Among heterosexual patients, length of stay did not differ between responders and nonresponders (*d* = 0.04); among SM patients nonresponders had a longer stay than responders (*d* = 0.69).

### Hopelessness

#### Intake-discharge differences

There was a close to zero difference in improvement of hopelessness between heterosexual and SM patients in the unadjusted (*d* = 0.03) and adjusted analysis (Table 3). Only F6 diagnosis (personality disorders) interacted significantly with sexual orientation: among heterosexual patients there was no difference in change of hopelessness between those with and without F6 diagnosis, whereas SM patients without F6 diagnosis showed slightly more improvement (*d* = 0.14) and SM patients without F6 diagnosis slightly less improvement (*d* = 0.26) than heterosexual patients.

#### Responder analysis

The odds for not responding to treatment were comparable between heterosexual and SM patients in the unadjusted analysis (*OR* = 1.25, 95%-*CI* 0.75–2.15). In the adjusted analysis, SM patients were somewhat more likely to be nonresponders compared to heterosexuals (*OR* = 2.01, 95%-*CI* 0.97–4.16) but the difference did not reach statistical significance (*p* = .06). Significant interactions with sexual orientation only occurred for F4 diagnosis (anxiety, dissociative, stress-related, somatoform disorders): among those without an F4 diagnosis, SM patients were more likely to be nonresponders than heterosexual patients (*OR* = 2.12), whereas among those with an F4 diagnosis the reverse association was found (*OR* = 0.51).

### Depression

#### Intake-discharge differences

There was a close to zero difference in improvement in depression between heterosexual and SM patients in the unadjusted model (*d* = 0.02). After adjusting for confounders, SM patients showed significantly more improvement than heterosexual patients (Table 3). Only length of stay interacted significantly with sexual orientation: for patients with a stay of maximum 14 days, SM patients had slightly greater improvement in depression than heterosexuals (*d* = 0.28), whereas among those with longer stays (> 30 days), SM patients demonstrated somewhat lesser improvement (*d* = 0.34).

#### Responder analysis

The odds for responding to treatment were comparable between heterosexual and SM patients in the unadjusted analysis (*OR* = 1.06, 95%-*CI* 0.67–1.63). In the adjusted analysis, SM patients were significantly less likely to be nonresponders, compared to heterosexuals (*OR* = 0.77, 95%-*CI* 0.46–1.28). Significant interactions with sexual orientation only occurred for length of stay: there was no sexual orientation difference among responders (*d* = 0.06), whereas among nonresponders, SM patients had longer stays than heterosexual patients (*d* = 0.73).

### Treatment expectancy and working alliance (secondary study aim)

#### Treatment expectancy

There was a close to zero difference (*d* = 0.02) between heterosexual and SM patients in the unadjusted analysis (Table 3). However, in the adjusted model, sexual orientation became a significant predictor (*p* = .046), meaning that SM patients had more positive treatment expectations than heterosexual patients. Only nationality interacted with sexual orientation: there were no sexual orientation differences in expectation among Austrians (*d* = 0.13), whereas SM non-Austrians scored somewhat lower in expectancy than heterosexual non-Austrians (*d* = 0.49).

#### Working alliance

There was a nonsignificant and very small (*d* = 0.09) difference between heterosexual and SM patients in the unadjusted analysis (Table 3). In the adjusted model, sexual orientation became a significant predictor (*p* = .02), meaning that SM patients had higher levels of working alliance compared to heterosexual patients. Only nationality and length of stay interacted with sexual orientation: There were only slight sexual orientation differences in working alliance among Austrians (*d* = 0.13), whereas SM non-Austrians scored much lower in working alliance levels than heterosexual non-Austrians (*d* = 0.82). With respect to treatment length, sexual orientation differences were small for short-term stays and disappeared for longer stays.

## Discussion

In our study, we compared SM and heterosexual patients at risk for suicide in a public psychiatric setting. The primary goal of our study was to compare treatment outcome by sexual orientation, and the secondary aim to compare treatment expectancy and working alliance by sexual orientation.

With respect to treatment outcome, contrary to our expectations, there were no notable differences by sexual orientation in all unadjusted analyses for change in suicide ideation, hopelessness and depression. No significant differences were found in the related responder analysis, either. The observed differences lacked statistical significance and were of small magnitude. When adjusting for potential confounders (sociodemographics, diagnosis, and length of stay), the sexual orientation differences became significant for depression, but contrary to the hypothesis: SM patients demonstrated larger improvements in levels of depression and a higher likelihood of responding.

Concerning our secondary study aim, we found that SM and heterosexual patients were comparable in their levels of treatment expectancy and working alliance. When adjusting for confounders, SM patients demonstrated significantly greater treatment expectation and better working alliance than heterosexual patients.

The results are surprising, given that previous studies reported barriers to healthcare for SM patients leading potentially to poorer treatment outcomes. There are several possible explanations for these findings, some of them related to potential limitations of our study.

It remains unknown if the CI-SP department is representative of other mental health departments with regard to SM-specific competencies and SM-affirmative attitudes among the staff. For example, an English study found that, overall, satisfaction with healthcare was lower for SM than heterosexual patients, but there was great variation between general practices, with some practices evaluated similarly by SM and heterosexual patients [29]. In the CI-SP department, the staff has not received any formal training in SM-specific competencies. However, some of the authors of this paper have published on suicide risk of SM individuals and are also members of the therapeutic team, with one of them being openly gay. This could have led to changes in attitudes and increased awareness about SM issues [70], thereby improving care for SM patients. Replication studies in other clinical settings are needed. Staff attitudes and expertise were not assessed in this study, but should be assessed in future studies.

An alternative explanation is related to the contextual model of psychotherapeutic change [36]. According to this model, treatment success results from contextual factors, including empathic and real therapeutic relationship, creation of positive expectations, and a shared working model of the problem. In contrast, specific ingredients, such as specific forms of therapies or adherence to protocols are of minor importance for treatment success. If SM-specific competencies constitute specific ingredients in therapy, then our results are not surprising. This alternative explanation is also supported by the finding that SM and heterosexual patients do not differ on two important common factors of treatment (working alliance and treatment expectation). Perhaps the lack of SM-specific competencies can be overcome by a strong therapeutic alliance, in the sense of “I had to educate my therapist about the realities of being a lesbian woman, but he/she was a kind person and really tried to help me.” In line with this argument, although a large majority of gay men (including those with major depression) reported that they would more likely present and speak openly in a gay-friendly treatment setting, only a plurality felt that gay-friendly providers would improve the treatment outcome [33]. However, there may also be a tradeoff at some point whereby the lack in SM competencies starts to have a negative impact on treatment success. A strong therapeutic alliance can mitigate disclosure of sexual orientation, but recognizing the heteronormative value system by the clinician is a key to establish a strong therapeutic relationship [31]. Furthermore, in psychotherapy, therapists who were perceived as helpful clearly had more SM-specific competencies than unhelpful therapists [34]. Future studies could use a similar methodology as Liddle’s [34] to study psychiatric settings.

The argument above could also be applied to the interesting finding that treatment expectation was comparable between SM and heterosexual patients. If SM patients had negative experiences in healthcare or in society in general, then they should have lower treatment expectancies than heterosexuals before the beginning of treatment. However, we assessed treatment expectancy after the intake interview, which is done in a patient-centered, narrative style known to enhance therapeutic alliance and outcome with suicidal patients [38, 71]. Thus, the intake interview itself could have improved treatment expectancy for SM patients, but this remains an issue for further empirical investigations.

Our findings are relevant both in terms of sexual orientation-based health disparities and mental health service provision. A wealth of literature [3, 16] outlines the myriad ways SM status is a risk factor for poor mental health and elevated suicide risk. While many approaches to health disparity reduction focus on specific cultural competence training (see [72] for review), our data suggest the potential for a common factors approach to reduce such disparities in an inpatient paradigm. Effective contextual factors are more easily and generally applicable to treatment settings, offering promise for further implementation and study.

### Limitations

Our results could lack validity through biased self-reports, a problem that is shared by all research on hidden populations. For example, some SM patients may have remained undisclosed in the assessment – i.e., identified as heterosexual or have chosen the “I don’t understand” option – or some may have refused to take part or complete the assessment. We did not use open-ended response options in the items on sexual behavior and attraction, and this may have been problematic for some people, for example those with lacking sexual attraction. The percentage of patients who identified as SM resembles results from the general US population [73] and was slightly lower than in a study of patients presenting at an emergency department [74], suggesting that underreporting may not be severe. However, given that SM individuals are at higher risk for suicide and mental disorders, they should be overrepresented among patients, but only if they use mental health services at least as frequently as heterosexuals. Nonetheless, underreporting is a reality, proven by a few patients who disclosed during treatment but refused to do so in the assessment. Coming out during treatment could interact with treatment outcome but we did not assess disclosure to the clinicians systematically. Furthermore, problems with coming out is a frequent cause of suicide attempts among gay men [75]; thus, underreporting and suicide risk are likely correlated among SM patients. Unfortunately, we could not systematically assess such misclassified individuals. This bias would be especially problematic if misclassification varies with the outcome variables – i.e., if those with low treatment expectancy or treatment success hide their SM status. Perhaps some of the bias can be reduced with follow-up studies whereby patients are assessed anonymously by a third party. Despite these limitations, we found the assessment of sexual orientation feasible, in line with a recent US study [74], and encourage measures of sexual orientation in assessment instruments, as recommended by the Institute of Medicine [16] in order to improve care for SM patients [76, 77].

Another limitation is that we did not assess transgender and intersex but only a dichotomized category of gender (male/female) which could again produce biases. Moreover, this is itself an example of lacking minority-specific competency, in this case about gender minorities which should be avoided. This has been corrected this in the meantime. We also did not have information of the type of medication for individual patients available for analysis. However, this would only be important if medication varies with sexual orientation or if medication works differently for SM than heterosexual patients, which is unlikely. Of course, medication varies with diagnoses and some diagnoses were disproportionately represented among SM patients. This is why we investigated all interaction effects with diagnosis and adjusted for diagnosis in the multivariate analysis.

The sample size limits the precise estimation for subgroups of SM individuals as well as subgroups based on gender, ethnic background, diagnosis, and other relevant factors. Additional analysis for the three dimensions of sexual orientation (provided upon request) suggest that the results for subgroups of SM individuals did not differ significantly, with a few exceptions that are contrary to our hypothesis. Bisexually behaving patients had higher levels of treatment expectation, same-sex attracted patients had higher levels of working alliance, other identified patients (most of them heterosexually classified) had less improvement in depression. These results could be false positives resulting from multiple testing, since nearly all differences are far from being significant in Tukey post hoc comparison tests (results not shown). On the other hand, given the small subsamples, even larger differences will not become statistically significant. The same applies to the interaction effects, where – out of the many gender or diagnosis interaction effects with sexual orientation – only a few were statistically significant. Of note, it seems that SM patients of non-Austrian nationality responded less optimally to treatment, and they also had lower treatment expectancy and working alliance, compared to SM Austrians. This finding deserves further exploration, since these results may be false positives, given the large number of interactions. Larger replication studies are needed to allow subgroup analysis and examine interaction effects.

We did not record if patients had had a history of prior hospitalization at the CI-SP department. If patients had been in the CI-SP department before, their previous experience with the treating health professionals might have influenced their current treatment expectation.

Despite these limitations, this study is, to our knowledge, the first to investigate treatment success and related variables in relation to sexual orientation in a sample of patients at risk for suicide in a general psychiatric hospital and thus has more ecological validity than previous studies.

## Conclusions

SM and heterosexual patients at risk for suicide in a public psychiatric hospital setting did not differ significantly in treatment outcome, treatment expectation, and working alliance. This is contrary to what is expected from the literature where barriers for SM patients are reported which have a negative impact on treatment outcome and satisfaction with treatment. Perhaps SM-specific competencies in our clinical setting were not representative (this was not assessed, unfortunately), or common factors of therapy can, to a certain degree, compensate for a lack of SM-specific competencies. Replications in other treatment settings are needed before these findings can be generalized.

## Additional file

Additional file 1: Supplement-additional-results-statistical-analysis-in-paper. (PDF 687 kb)

## Abbreviations

95%-*CI*: 95-Percent confidence interval
CI-SP: Crisis intervention and suicide prevention
SM: Sexual minority
