# Supplementary material for: Sexual orientation differences in treatment expectation, alliance, and outcome among patients at risk for suicide in a public psychiatric hospital
Source: BMC Psychiatry. 2017 May 15;17:184. doi: 10.1186/s12888-017-1337-8 (PMC5433065; doi:10.1186/s12888-017-1337-8)

Online Supplement

Detailed Results for Analyses in Paper

For paper "Sexual Orientation Differences in Treatment Expectation, Alliance, and Outcome. Findings from Patients at Risk for Suicide in a Public Psychiatric Hospital"

Content

1 Comments to Statistical Analysis ... 3
2 Treatment Outcome – Difference Intake-Discharge Assessments ... 4
2.1 Change of Suicide Ideation ... 4
2.1.1 Interaction with Confounders ... 4
2.1.2 Adjusting for Confounders ... 5
2.2 Change of Hopelessness ... 7
2.2.1 Interaction with Confounders ... 7
2.2.2 Adjusting for Confounders ... 8
2.3 Change of Depression ... 10
2.3.1 Interaction with Confounders ... 10
2.3.2 Adjusting for Confounders ... 11
3 Responder Analysis ... 13
3.1 Suicide Ideation ... 13
3.1.1 Interactions with Confounders ... 14
3.1.2 Adjusting for Confounders ... 16
3.2 Hopelessness ... 18
3.2.1 Interactions with Confounders ... 18
3.2.2 Adjusting for Confounders ... 19
3.3 Depression ... 21
3.3.1 Interactions with Confounders ... 21
3.3.2 Adjusting for Confounders ... 22
4 Treatment Expectancy ... 24
3.4 Interaction with Confounders ... 24
3.4.1 Nationality ... 24
3.5 Adjusting for Confounders ... 25
5 Working Alliance ... 26
3.6 Interaction with Confounders ... 26
3.7 Adjusting for Confounders ... 28

1 Comments to Statistical Analysis

These supplemental statistical results are carried out with R 3.1.3 using additional packages suc as “psych” and “rms” (for some descriptive tabulation), ggplot2 (for interaction plots), and “foreign” (for importing SPSS files).

2 Treatment Outcome – Difference Intake-Discharge Assessments

2.1 Change of Suicide Ideation

Linear Regression – SM Status as only predictor

```
Residuals:
    Min      1Q  Median      3Q     Max 
-28.797  -4.797  -3.305   5.203  27.203 

Coefficients:
            Estimate Std. Error t value Pr(>|t|)    
(Intercept)   4.7968     0.3475  13.804   <2e-16 ***
smyes         0.5085     0.7639   0.666    0.506    
---
Signif. codes:  0 ‘***’ 0.001 ‘**’ 0.01 ‘*’ 0.05 ‘.’ 0.1 ‘ ’ 1

Residual standard error: 7.786 on 631 degrees of freedom
Multiple R-squared:  0.0007019,	Adjusted R-squared:  -0.0008818 
F-statistic: 0.4432 on 1 and 631 DF,  p-value: 0.5058
```

2.1.1 Interaction with Confounders

F7 Diagnosis

```
lm(formula = d_BSI ~ sm * F7)

Residuals:
   Min     1Q Median     3Q    Max 
-28.83  -4.83  -3.00   5.17  27.17 

Coefficients:
            Estimate Std. Error t value Pr(>|t|)    
(Intercept)   4.8298     0.3559  13.569   <2e-16 ***
smyes         0.1702     0.7780   0.219    0.827    
F7yes        -0.6375     1.5640  -0.408    0.684    
smyes:F7yes   8.6375     3.8711   2.231    0.026 *  
---
Signif. codes:  0 ‘***’ 0.001 ‘**’ 0.01 ‘*’ 0.05 ‘.’ 0.1 ‘ ’ 1

Residual standard error: 7.766 on 629 degrees of freedom
Multiple R-squared:  0.009005,	Adjusted R-squared:  0.004278 
F-statistic: 1.905 on 3 and 629 DF,  p-value: 0.1275
```

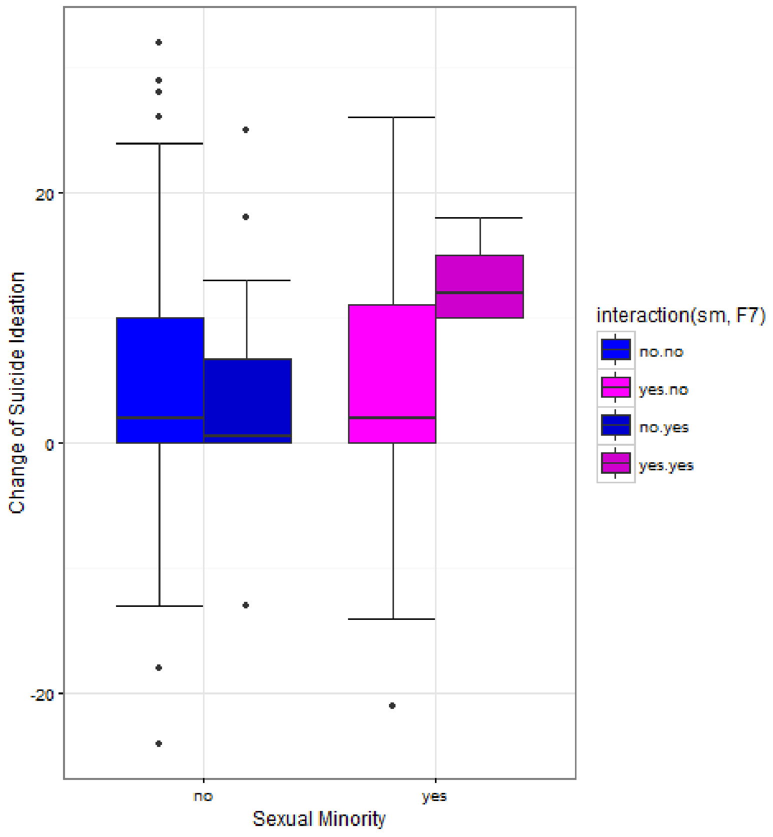

2.1.2 Adjusting for Confounders

Full Multivariate Model

```
Call:
lm(formula = d_BSI ~ nationality + F3 + F7*sm + log(length_stay) * sm)

Residuals:
    Min      1Q  Median      3Q     Max 
-26.363  -5.141  -2.676   4.662  30.568 

Coefficients:
                      Estimate Std. Error t value Pr(>|t|)   
(Intercept)             3.2972     2.1011   1.569  0.11710   
nationality            -1.6587     0.9499  -1.746  0.08127 . 
F3yes                   2.1572     0.7224   2.986  0.00293 **
F7yes                  -0.5045     1.5524  -0.325  0.74528   
smyes                   2.2559     3.6490   0.618  0.53666   
log(length_stay)        0.5845     0.5730   1.020  0.30806   
F7yes:smyes             8.1731     3.8412   2.128  0.03375 * 
smyes:log(length_stay) -0.6770     1.1548  -0.586  0.55790   
---
Signif. codes:  0 ‘***’ 0.001 ‘**’ 0.01 ‘*’ 0.05 ‘.’ 0.1 ‘ ’ 1

Residual standard error: 7.701 on 625 degrees of freedom
Multiple R-squared:  0.0317,	Adjusted R-squared:  0.02086 
F-statistic: 2.923 on 7 and 625 DF,  p-value: 0.00512
```

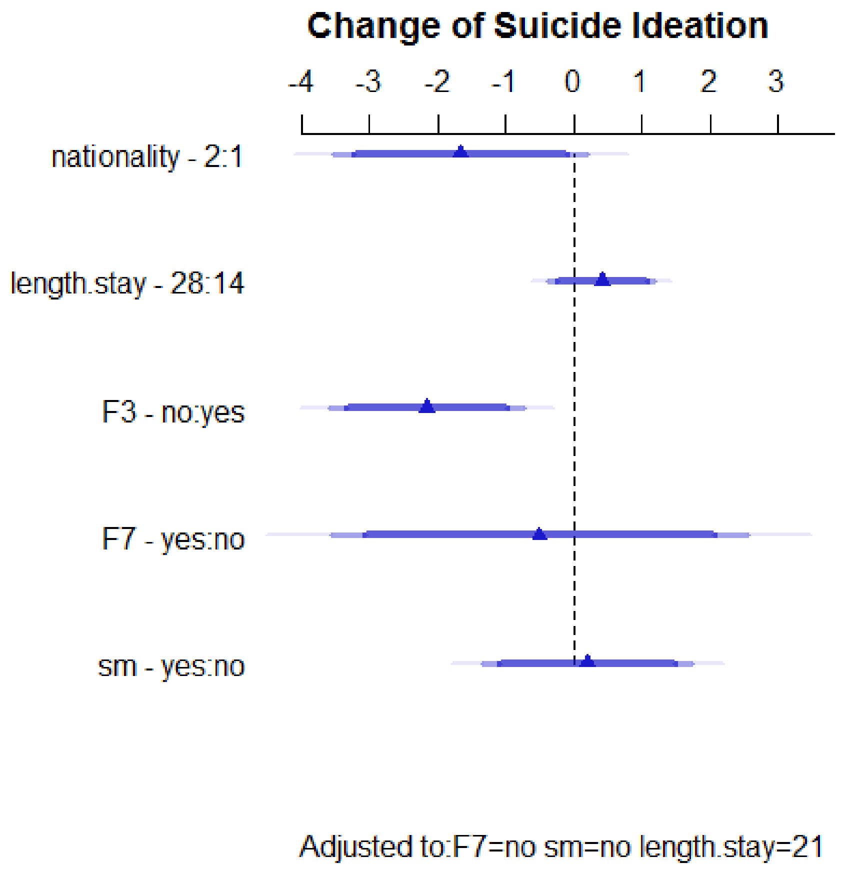
Change of Suicide Ideation
-4
-3
-2
-1
0
1
2
3
nationality - 2:1
length.stay - 28:14
F3 - no:yes
F7 - yes:no
sm - yes:no
Adjusted to:F7=no sm=no length.stay=21

2.2 Change of Hopelessness

Linear Regression – SM Status as only predictor

```
Residuals:
    Min      1Q  Median      3Q     Max 
-28.797  -4.797  -3.305   5.203  27.203 

Coefficients:
            Estimate Std. Error t value Pr(>|t|)    
(Intercept)   4.7968     0.3475  13.804   <2e-16 ***
smyes         0.5085     0.7639   0.666    0.506    
---
Signif. codes:  0 ‘***’ 0.001 ‘**’ 0.01 ‘*’ 0.05 ‘.’ 0.1 ‘ ’ 1

Residual standard error: 7.786 on 631 degrees of freedom
Multiple R-squared:  0.0007019,	Adjusted R-squared:  -0.0008818 
F-statistic: 0.4432 on 1 and 631 DF,  p-value: 0.5058
```

2.2.1 Interaction with Confounders

F6 Diagnosis

```
lm(formula = d_BHS ~ sm * F6)

Residuals:
     Min       1Q   Median       3Q      Max 
-13.5024  -3.5024  -0.5024   2.7634  15.4976 

Coefficients:
            Estimate Std. Error t value Pr(>|t|)    
(Intercept)   3.5024     0.2378  14.726   <2e-16 ***
smyes         0.7341     0.5542   1.325   0.1858    
F6yes         0.2420     0.5617   0.431   0.6667    
smyes:F6yes  -2.1891     1.0860  -2.016   0.0442 *  
---
Signif. codes:  0 ‘***’ 0.001 ‘**’ 0.01 ‘*’ 0.05 ‘.’ 0.1 ‘ ’ 1

Residual standard error: 4.828 on 629 degrees of freedom
Multiple R-squared:  0.00733,	Adjusted R-squared:  0.002596 
F-statistic: 1.548 on 3 and 629 DF,  p-value: 0.2009
```

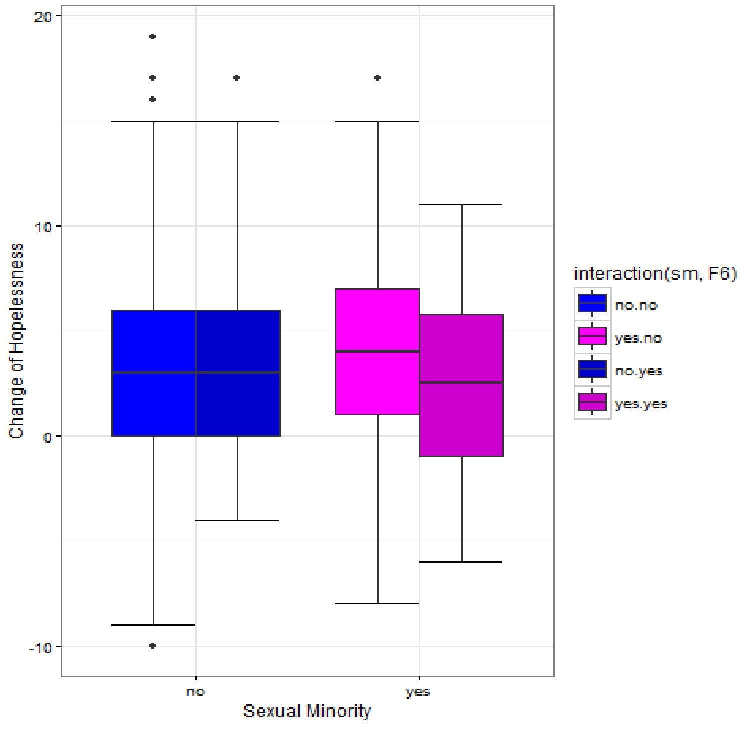

2.2.2 Adjusting for Confounders

Full Multivariate Model

```
Call:
lm(formula = d_BHS ~ income     + m_languag + F0 + F3 + F8 +
    F6 * sm + log(length_stay))

Residuals:
     Min       1Q   Median       3Q      Max
-13.0885  -3.3232  -0.1569   3.0194  16.0740

Coefficients:
                  Estimate Std. Error t value Pr(>|t|)
(Intercept)      4.3594531  1.2343305   3.532 0.000443 ***
income           0.0004242  0.0001659   2.556 0.010811 *
m_languag       -1.6542639  0.6420080  -2.577 0.010204 *
F0yes            1.6797839  1.0501397   1.600 0.110198
F3yes            0.8831951  0.4548372   1.942 0.052614 .
F8yes           -2.0280383  1.1485324  -1.766 0.077925 .
F6yes            0.6082591  0.5630221   1.080 0.280406
smyes            0.8180816  0.5514322   1.484 0.138432
log(length_stay)-0.1074547  0.3098608  -0.347 0.728871
F6yes:smyes     -2.3064893  1.0792971  -2.137 0.032984 *
---
Signif. codes:  0 ‘***’ 0.001 ‘**’ 0.01 ‘*’ 0.05 ‘.’ 0.1 ‘ ’ 1

Residual standard error: 4.76 on 623 degrees of freedom
Multiple R-squared:  0.04391,  Adjusted R-squared:  0.0301
F-statistic: 3.179 on 9 and 623 DF,  p-value: 0.0009075
```

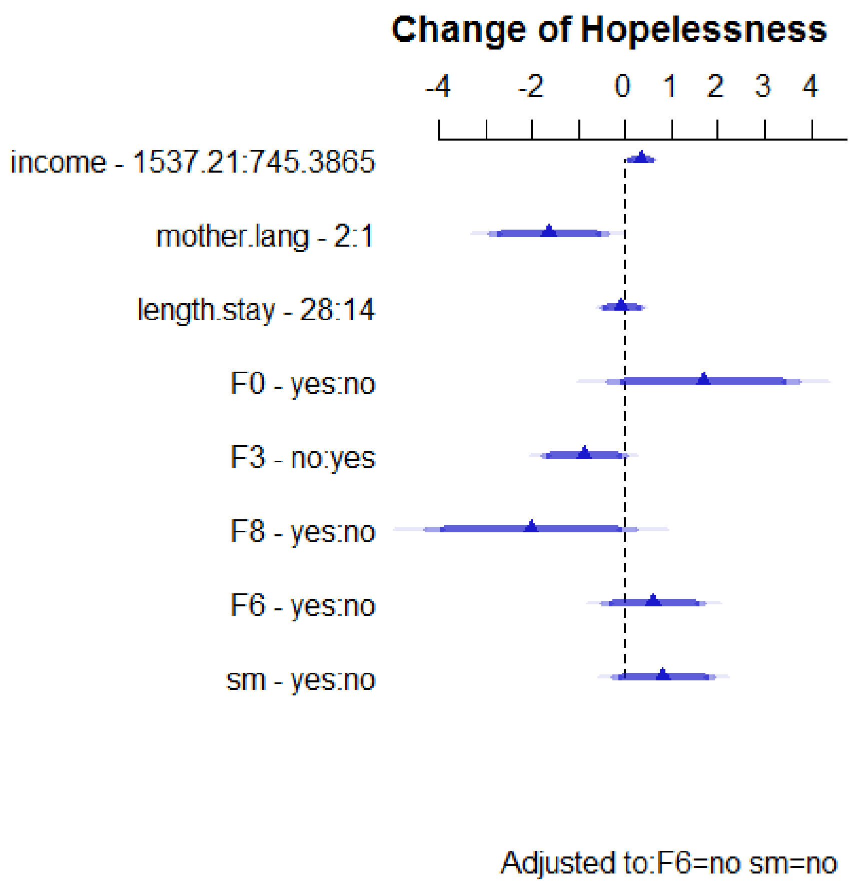
Change of Hopelessness
-4
-2
0
1
2
3
4
income - 1537.21:745.3865
mother.lang - 2:1
length.stay - 28:14
F0 - yes:no
F3 - no:yes
F8 - yes:no
F6 - yes:no
sm - yes:no
Adjusted to:F6=no sm=no

2.3 Change of Depression

Linear Regression – SM Status as only predictor

```
Residuals:
     Min       1Q   Median       3Q      Max
-13.5458  -3.5458  -0.5458   2.4542  15.4542

Coefficients:
            Estimate Std. Error t value Pr(>|t|)
(Intercept)   3.5458     0.2159  16.423   <2e-16 ***
smyes         0.1259     0.4746   0.265    0.791
---
Signif. codes:  0 ‘***’ 0.001 ‘**’ 0.01 ‘*’ 0.05 ‘.’ 0.1 ‘ ’ 1

Residual standard error: 4.837 on 631 degrees of freedom
Multiple R-squared:  0.0001116,       Adjusted R-squared:  -0.001473
F-statistic: 0.07042 on 1 and 631 DF,  p-value: 0.7908
```

2.3.1 Interaction with Confounders

Length of stay

```
lm(formula = d_BDI ~ sm * length_stay)

Residuals:
    Min      1Q  Median      3Q     Max
-29.366  -7.309  -0.534   6.677  34.391

Coefficients:
                 Estimate Std. Error t value Pr(>|t|)
(Intercept)      12.16639    0.67357  18.063   <2e-16 ***
smyes             2.67140    1.42736   1.872   0.0617 .
length_stay       0.01427    0.01957   0.729   0.4664
smyes:length_stay-0.08287    0.03400  -2.437   0.0151 *
---
Signif. codes:  0 ‘***’ 0.001 ‘**’ 0.01 ‘*’ 0.05 ‘.’ 0.1 ‘ ’ 1

Residual standard error: 10.78 on 629 degrees of freedom
Multiple R-squared:  0.01051,  Adjusted R-squared:  0.005794
F-statistic: 2.228 on 3 and 629 DF,  p-value: 0.08382
```

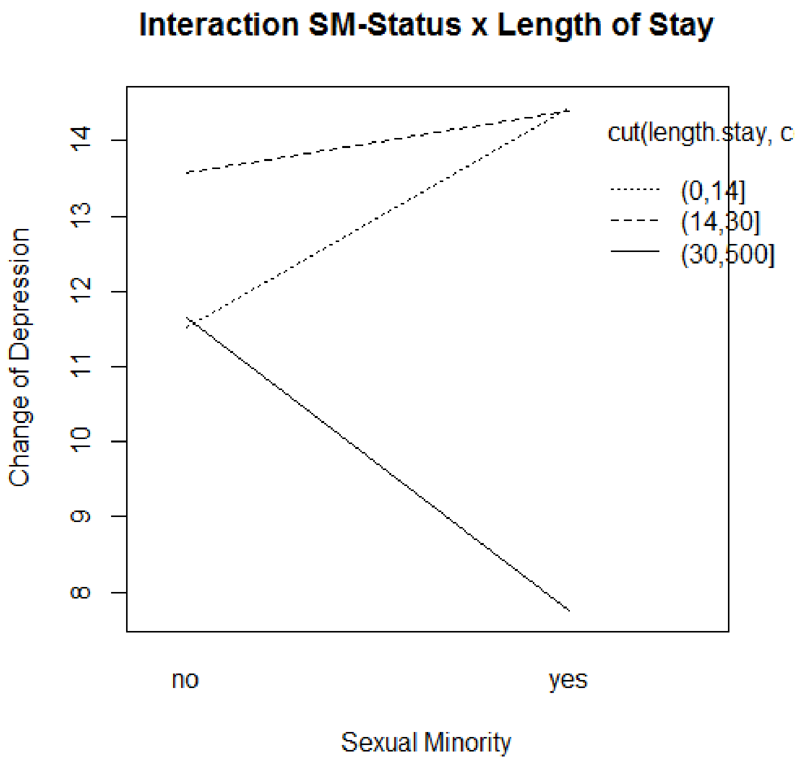

SM vs. heterosexuals:Lengh of Stay < 15 days d = - 0.28
> 30 days d = 0.34

2.3.2 Adjusting for Confounders

Full Multivariate Model

```
Call:
lm(formula = d_BDI ~ income     + m_languag + F3 + log(length_stay) *
    sm)

Residuals:
    Min      1Q  Median      3Q     Max
-30.992  -7.020  -0.964   6.579  33.256

Coefficients:
                        Estimate Std. Error t value Pr(>|t|)
(Intercept)           14.1500874  2.9502233   4.796 2.02e-06 ***
income                 0.0007955  0.0003645   2.182  0.02946 *
m_languag             -4.5701916  1.4198232  -3.219  0.00135 **
F3yes                  3.9406123  0.9907661   3.977  7.78e-05 ***
log(length_stay)      -0.2130309  0.7837679  -0.272  0.78586
smyes                 11.7819455  4.9793312   2.366  0.01828 *
log(length_stay):smyes-3.6224037  1.5779521  -2.296  0.02203 *
---
Signif. codes:  0 ‘***’ 0.001 ‘**’ 0.01 ‘*’ 0.05 ‘.’ 0.1 ‘ ’ 1

Residual standard error: 10.54 on 626 degrees of freedom
Multiple R-squared:  0.05895,  Adjusted R-squared:  0.04993
F-statistic: 6.536 on 6 and 626 DF,  p-value: 1.046e-06
```

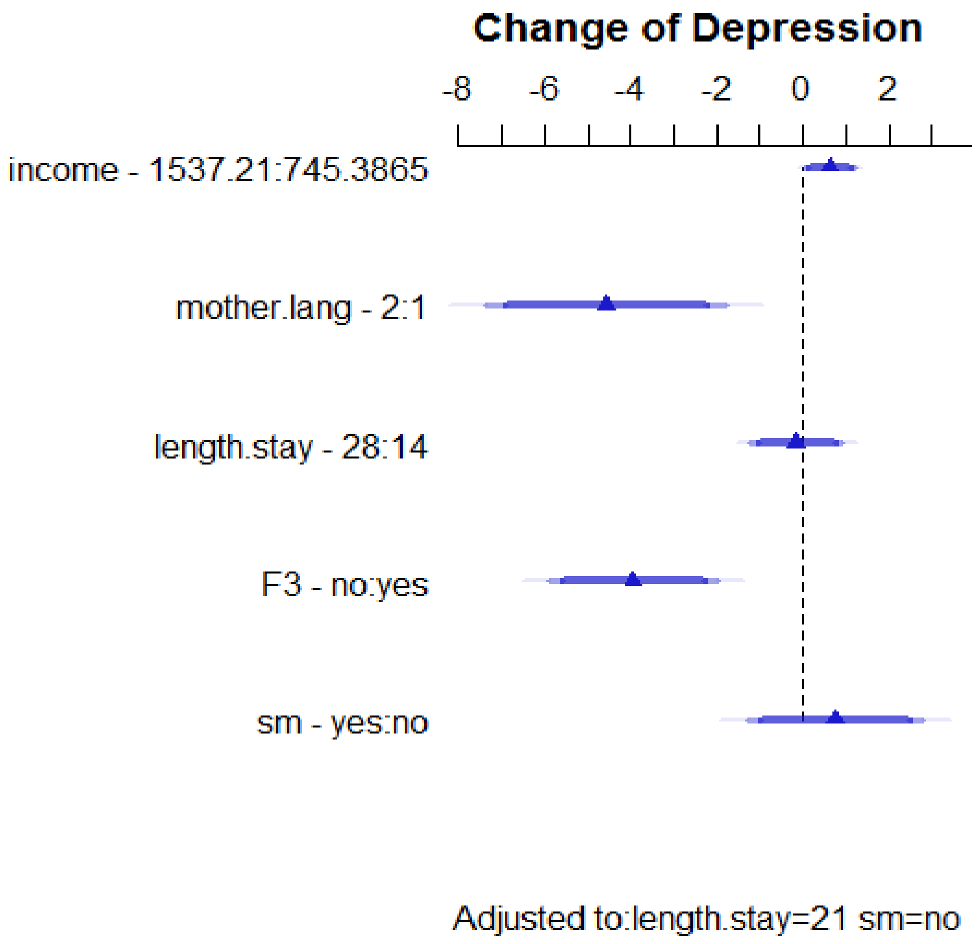
Change of Depression
-8
-6
-4
-2
0
2
income - 1537.21:745.3865
mother.lang - 2:1
length.stay - 28:14
F3 - no:yes
sm - yes:no
Adjusted to:length.stay=21 sm=no

3 Responder Analysis

Procedure accoding to Hiller, W., Schindler, A.C. & Lambert, M. J. (2011). Defining response and remission in psychotherapy research: A comparison of the RCI and the method of percent improvement. *Psychotherapy Research, 22*, 1 – 11.

Responder if:

1. baseline is in the pathological range and there is at least 50% improvement in the clinical range (baseline minus cut-off). For example, if a patient scores 30 on the BDI at baseline 20 at follow-up (10-point difference), the cut-off is 14+, then this results in a percentual improvement of 10 / (30-14) * 100 = 62.5%
2. baseline is in the pathological range and the overall improvement has to be at least 25% in the general range (because if a patient's baseline is only slightly above the cut-off, then it is too easy to gain > 50% possible change in the clinical range). E.g, if a patient's baseline is 16 on the BDI and 14 after treatment, then this is an overall improvement of (16-14)/16*100 = 12.5% (but it would be 100% with criterion 1).
3. Patients can only be responders or nonresponders if the baseline is in the pathological range.

3.1 Suicide Ideation

Cutoff > 4 (arbitrary)

```
  res_BSI
sm    1. responder 2. nonresponder 3. nonpathological
  no           194              74                234
  yes           57              26                 48
```

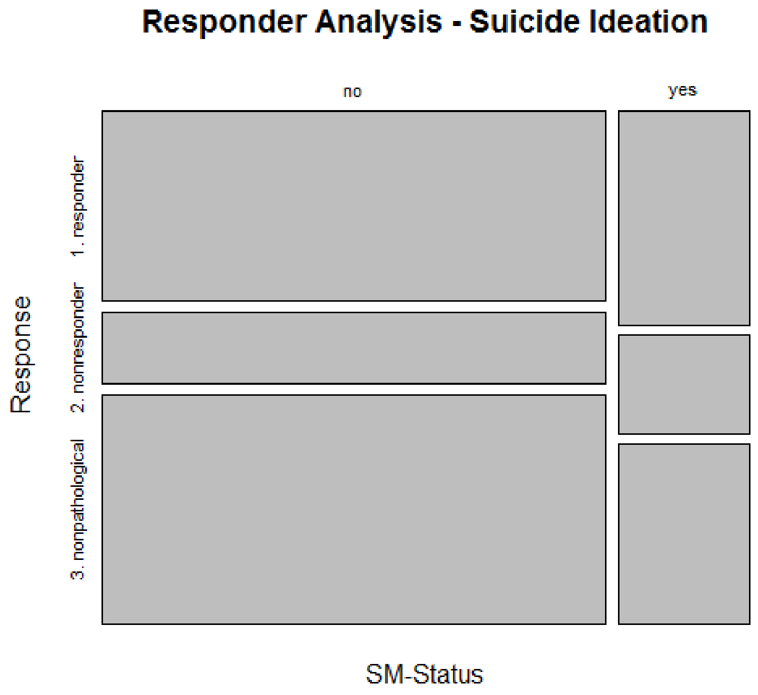


```
                Pearson's Chi-squared test
data:  sm and res_BSI
X-squared = 4.6542, df = 2, p-value = 0.09758
```

3.1.1 Interactions with Confounders

F3 Diagnosis

```
lrm(formula = res_BSI_bin ~ sm * F3)

                       Model Likelihood     Discrimination    Rank Discrim.
                          Ratio Test           Indexes           Indexes
Obs              351    LR chi2      9.51    R2       0.038    C       0.563
 1. responder    251    d.f.            3    g        0.236    Dxy     0.126
 2. nonresponder 100    Pr(> chi2) 0.0232    gr       1.267    gamma   0.221
max |deriv|    2e-11                         gp       0.052    tau-a   0.052
                                             Brier    0.198

                 Coef    S.E.   Wald Z Pr(>|Z|)
Intercept        -0.8473 0.3086 -2.75  0.0060
sm=yes            1.2993 0.5736  2.27  0.0235
F3=yes           -0.1441 0.3442 -0.42  0.6755
sm=yes * F3=yes  -1.5119 0.6625 -2.28  0.0225
```

```
        Oddsratio without F3: 3.56 (1.16-11.67) (SM more likely nonresp.)
        Oddsratio with F3: 0.81 (0.41-1.53)
```

F6 Diagnosis

```
Logistic Regression Model

lrm(formula = res_BSI_bin ~ sm * F6)
Frequencies of Missing Values Due to Each Variable

                       Model Likelihood     Discrimination    Rank Discrim.
                          Ratio Test           Indexes           Indexes
Obs              351    LR chi2     12.59    R2       0.051    C       0.594
 1. responder    251    d.f.            3    g        0.366    Dxy     0.187
 2. nonresponder 100    Pr(> chi2) 0.0056    gr       1.442    gamma   0.301
max |deriv|    1e-12                         gp       0.077    tau-a   0.077
                                             Brier    0.196

                 Coef    S.E.   Wald Z Pr(>|Z|)
Intercept        -1.0269 0.1601 -6.42  <0.0001
sm=yes           -0.4547 0.3852 -1.18  0.2378
F6=yes            0.2428 0.3082  0.79  0.4308
sm=yes * F6=yes   1.4464 0.5976  2.42  0.0155
```

```
        Oddsratio without F6: 0.64 (0.29-1.33) (SM less likely nonresp.)
        Oddsratio with F6: 2.65 (1.08-1.68)
```

Length of Stay

```
lrm(formula = res_BSI_bin ~ sm * length_stay)

                       Model Likelihood     Discrimination     Rank Discrim.
                          Ratio Test            Indexes           Indexes
Obs               351    LR chi2     10.76    R2       0.043    C       0.564
 1. responder     251    d.f.            3    g        0.224    Dxy     0.128
 2. nonresponder  100    Pr(> chi2) 0.0131    gr       1.252    gamma   0.145
max |deriv|     3e-04                         gp       0.042    tau-a   0.052
                                              Brier    0.197

                     Coef    S.E.   Wald Z Pr(>|Z|)
Intercept            -0.9977 0.1787 -5.58  <0.0001
sm=yes               -0.6452 0.4533 -1.42  0.1546
length_stay           0.0012 0.0042  0.30  0.7662
sm=yes * length_stay  0.0241 0.0115  2.09  0.0370
```

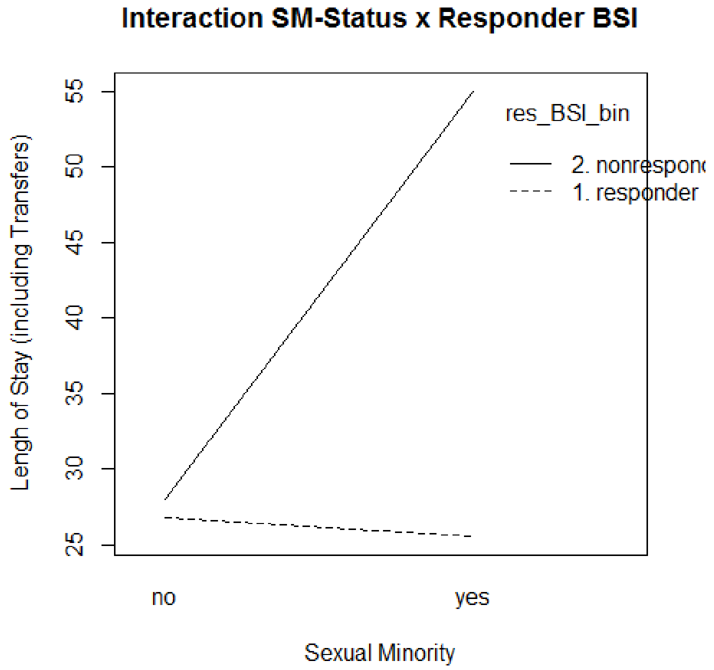

Cohen d responders 0.04 vs. nonresponders 0.69

3.1.2 Adjusting for Confounders

Full model

```
lrm(formula = res_BSI_bin ~ geschlecht + m_languag + F3 * sm +
    F6 * sm + log(length_stay) * sm)
Frequencies of Missing Values Due to Each Variable
res_BSI_bin  geschlecht     m_languag          F3          sm          F6
length_stay
        282           0             0           0           0           0           0


                        Model Likelihood     Discrimination    Rank Discrim.
                           Ratio Test           Indexes           Indexes
Obs              351    LR chi2     31.60    R2       0.123    C       0.675
 1. responder    251    d.f.            9    g        0.738    Dxy     0.351
 2. nonresponder 100    Pr(> chi2) 0.0002    gr       2.092    gamma   0.354
max |deriv|    3e-07                         gp       0.138    tau-a   0.143
                                             Brier    0.185

                      Coef    S.E.   Wald Z Pr(>|Z|)
Intercept             -3.5367 1.0410 -3.40  0.0007
geschlecht             0.5224 0.2637  1.98  0.0476
m_languag              0.7328 0.3605  2.03  0.0421
F3=yes                -0.0887 0.3639 -0.24  0.8074
sm=yes                -2.8614 1.8020 -1.59  0.1123
F6=yes                 0.1745 0.3233  0.54  0.5893
length_stay            0.3065 0.2259  1.36  0.1749
F3=yes * sm=yes       -1.0060 0.7764 -1.30  0.1951
sm=yes * F6=yes        0.9439 0.6868  1.37  0.1694
sm=yes * length_stay 0.9976 0.5269  1.89  0.0583


             Effects              Response : res_BSI_bin

 Factor       Low High Diff. Effect    S.E.    Lower 0.95 Upper 0.95
 geschlecht    1    2    1     0.522420 0.26368  0.0056113 1.039200
  Odds Ratio   1    2    1     1.686100      NA  1.0056000 2.827000
 m_languag     1    2    1     0.732840 0.36049  0.0263010 1.439400
  Odds Ratio   1    2    1     2.081000      NA  1.0267000 4.218100
 length_stay 14   28   14      0.212430 0.15659 -0.0944910 0.519350
  Odds Ratio 14   28   14      1.236700      NA  0.9098400 1.680900
 F3 - no:yes   2    1   NA     0.088744 0.36395 -0.6245800 0.802060
  Odds Ratio   2    1   NA     1.092800      NA  0.5354900 2.230100
 sm - yes:no   1    2   NA    -0.830190 0.44766 -1.7076000 0.047195
  Odds Ratio   1    2   NA     0.435970      NA  0.1813000 1.048300
 F6 - yes:no   1    2   NA     0.174530 0.32327 -0.4590600 0.808120
  Odds Ratio   1    2   NA     1.190700      NA  0.6318800 2.243700

Adjusted to: F3=yes sm=no F6=no length_stay=21
```

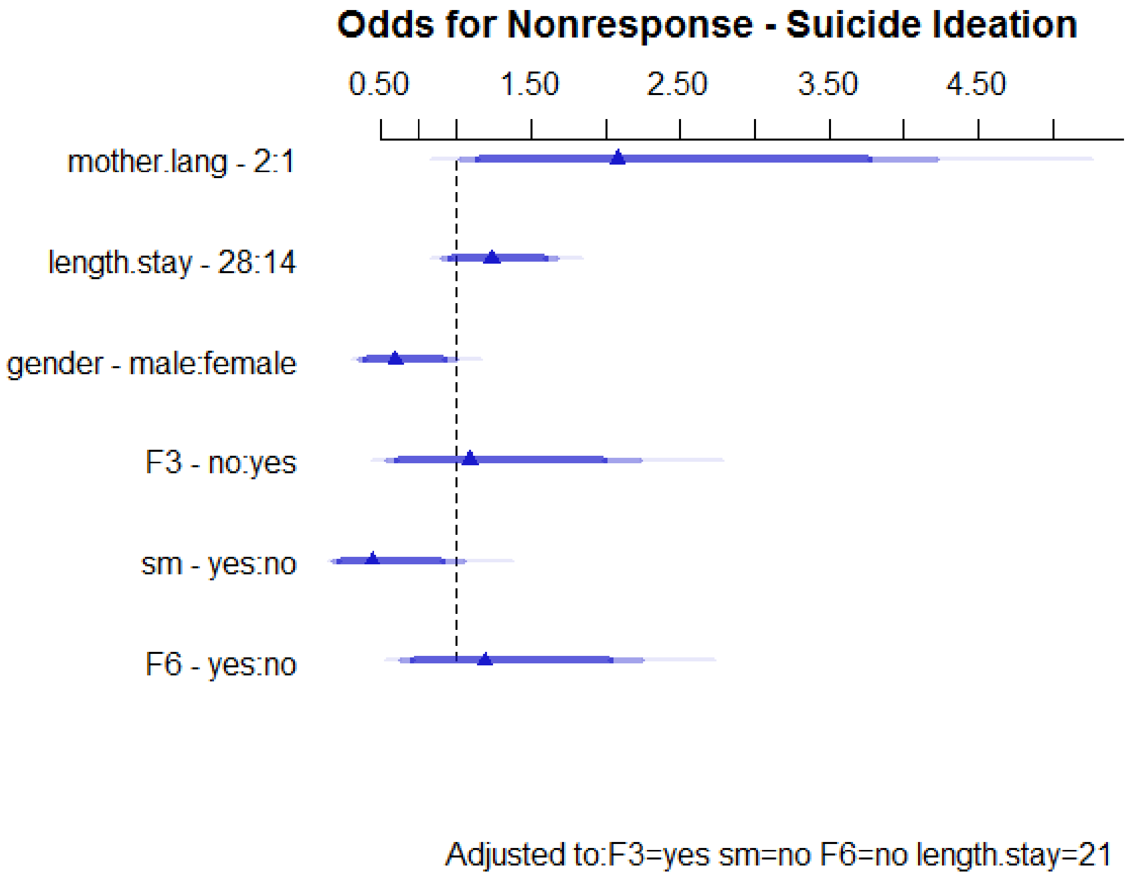
Odds for Nonresponse - Suicide Ideation
0.50
1.50
2.50
3.50
4.50
mother.lang - 2:1
length.stay - 28:14
gender - male:female
F3 - no:yes
sm - yes:no
F6 - yes:no
Adjusted to:F3=yes sm=no F6=no length.stay=21

3.2 Hopelessness

```
  res_BHS
sm    1. responder 2. nonresponder 3. nonpathological
  no              93           290               119
  yes             22            86                23
```

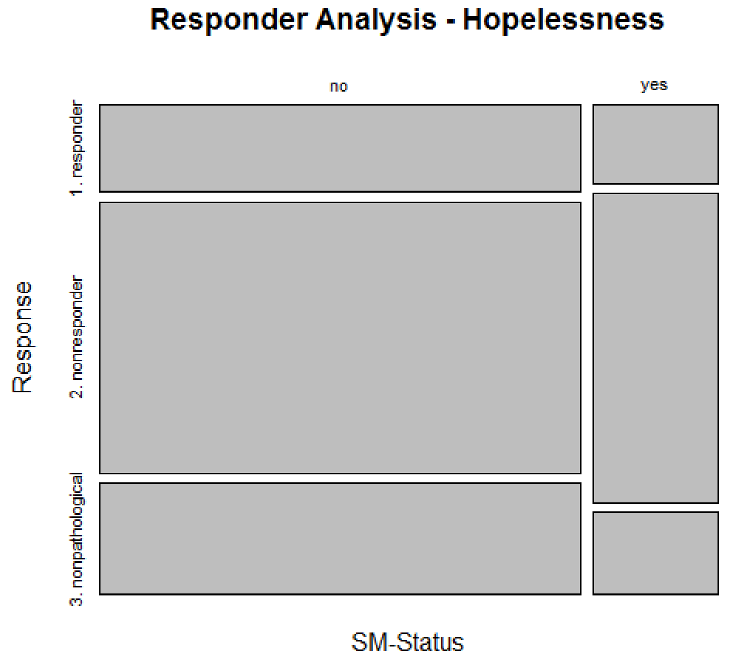


```
               Pearson's Chi-squared test
data:  sm and res_BHS
X-squared = 3.008, df = 2, p-value = 0.2222
```

3.2.1 Interactions with Confounders

F4 Diagnosis

```
lrm(formula = res_BHS_bin ~ sm * F4)

                        Model Likelihood     Discrimination    Rank Discrim.
                          Ratio Test            Indexes           Indexes
Obs               491    LR chi2      7.95    R2       0.024    C       0.568
 1. responder     115    d.f.            3    g        0.292    Dxy     0.136
 2. nonresponder 376    Pr(> chi2) 0.0470    gr       1.340    gamma   0.222
max |deriv|     4e-10                        gp       0.049    tau-a   0.049
                                             Brier    0.177

                 Coef    S.E.   Wald Z Pr(>|Z|)
Intercept         1.1036 0.1410  7.83  <0.0001
sm=yes            0.7682 0.3678  2.09  0.0367
F4=yes            0.1157 0.2640  0.44  0.6613
sm=yes * F4=yes -1.4279 0.5622 -2.54  0.0111

        Oddsratio without F4: 2.12 (1.07-4.64) (SM more likely nonresp.)
        Oddsratio with F4: 0.51 (0.23-1.22)
```

3.2.2 Adjusting for Confounders

Full model

```
Logistic Regression Model

lrm(formula = res_BHS_bin ~ nationality + F0 + F4 * sm + log(length_stay))
Frequencies of Missing Values Due to Each Variable
  res_BHS_bin nationality           F0           F4           sm    length_stay
          142           0            0            0            0              0


                      Model Likelihood     Discrimination     Rank Discrim.
                         Ratio Test           Indexes            Indexes
Obs               491   LR chi2      17.35  R2       0.052    C       0.606
 1. responder     115   d.f.             6  g        0.499    Dxy     0.213
 2. nonresponder 376   Pr(> chi2) 0.0081   gr       1.647    gamma   0.216
max |deriv|    5e-09                        gp       0.086    tau-a   0.076
                                            Brier    0.173

                 Coef    S.E.   Wald Z Pr(>|Z|)
Intercept        -0.4853 0.7244 -0.67  0.5029
nationality       0.5947 0.3874  1.54  0.1248
F0=yes           -1.1124 0.5380 -2.07  0.0387
F4=yes            0.1624 0.2688  0.60  0.5456
sm=yes            0.6983 0.3710  1.88  0.0598
length_stay       0.3213 0.1856  1.73  0.0834
F4=yes * sm=yes -1.4914 0.5672 -2.63  0.0086


             Effects              Response : res_BHS_bin

 Factor         Low High Diff. Effect   S.E.    Lower 0.95 Upper 0.95
 nationality     1   2    1     0.59466 0.38738 -0.164580   1.353900
  Odds Ratio     1   2    1     1.81240      NA  0.848250   3.872500
 length_stay    14  28   14     0.22269 0.12862 -0.029413   0.474780
  Odds Ratio    14  28   14     1.24940      NA  0.971010   1.607700
 F0 - yes:no     1   2   NA    -1.11240 0.53798 -2.166800  -0.057957
  Odds Ratio     1   2   NA     0.32877      NA  0.114540   0.943690
 F4 - yes:no     1   2   NA     0.16244 0.26877 -0.364340   0.689220
  Odds Ratio     1   2   NA     1.17640      NA  0.694650   1.992200
 sm - yes:no     1   2   NA     0.69832 0.37100 -0.028819   1.425500
  Odds Ratio     1   2   NA     2.01040      NA  0.971590   4.159800

Adjusted to: F4=no sm=no
```

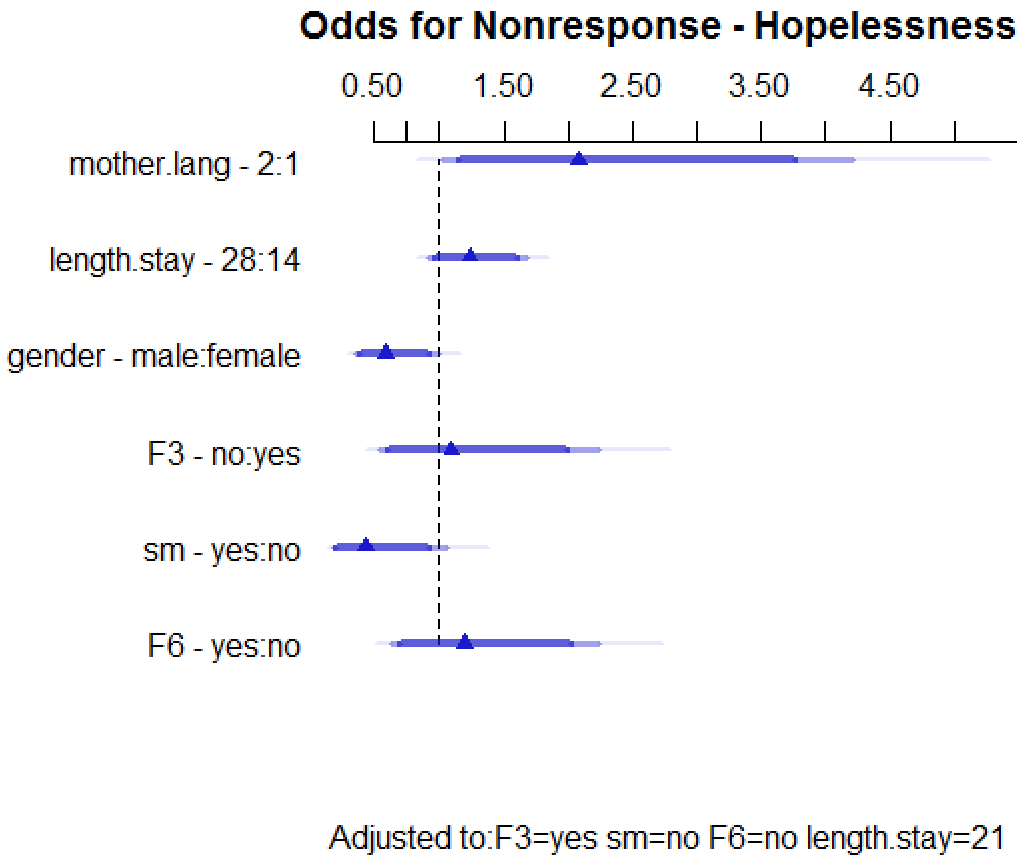
Odds for Nonresponse - Hopelessness
0.50
1.50
2.50
3.50
4.50
mother.lang - 2:1
length.stay - 28:14
gender - male:female
F3 - no:yes
sm - yes:no
F6 - yes:no
Adjusted to:F3=yes sm=no F6=no length.stay=21

3.3 Depression

```
    res_BDI
sm    1. responder 2. nonresponder 3.nonpathological
  no           304            127                71
  yes           84             37                10
```

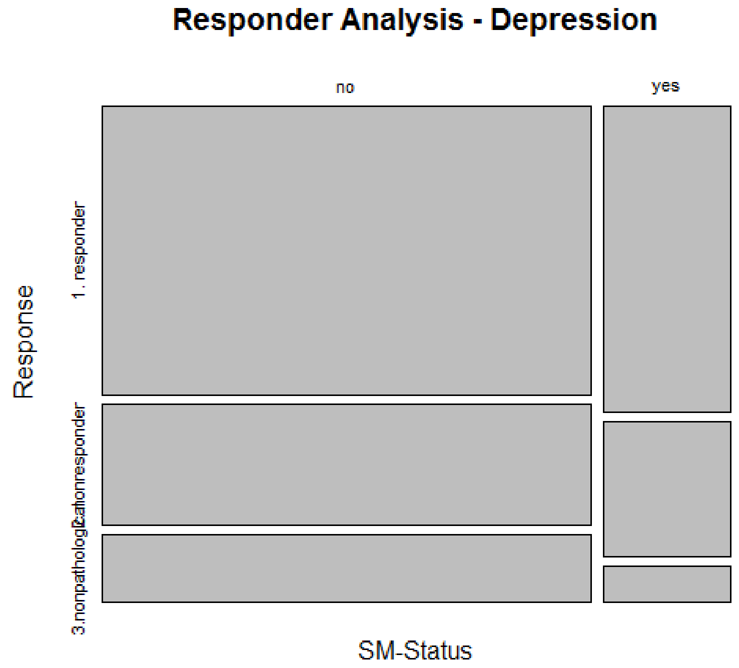


```
        Pearson's Chi-squared test

data:  sm and res_BDI
X-squared = 4.0038, df = 2, p-value = 0.1351
```

3.3.1 Interactions with Confounders

Length of Stay

```
lrm(formula = res_BDI_bin ~ sm * length_stay)
Frequencies of Missing Values Due to Each Variable
res_BDI_bin          sm  length_stay
         81           0            0

                      Model Likelihood     Discrimination    Rank Discrim.
                         Ratio Test           Indexes           Indexes
Obs              552    LR chi2     15.34    R2       0.039    C       0.584
 1. responder    388    d.f.            3    g        0.240    Dxy     0.168
 2. nonresponder 164    Pr(> chi2) 0.0015    gr       1.271    gamma   0.196
max |deriv|    3e-08                         gp       0.043    tau-a   0.070
                                             Brier    0.203

                     Coef    S.E.   Wald Z Pr(>|Z|)
Intercept            -0.9010 0.1448 -6.22  <0.0001
sm=yes               -0.8932 0.4087 -2.19  0.0288
length_stay           0.0011 0.0039  0.29  0.7748
sm=yes * length_stay  0.0315 0.0121  2.59  0.0095
```

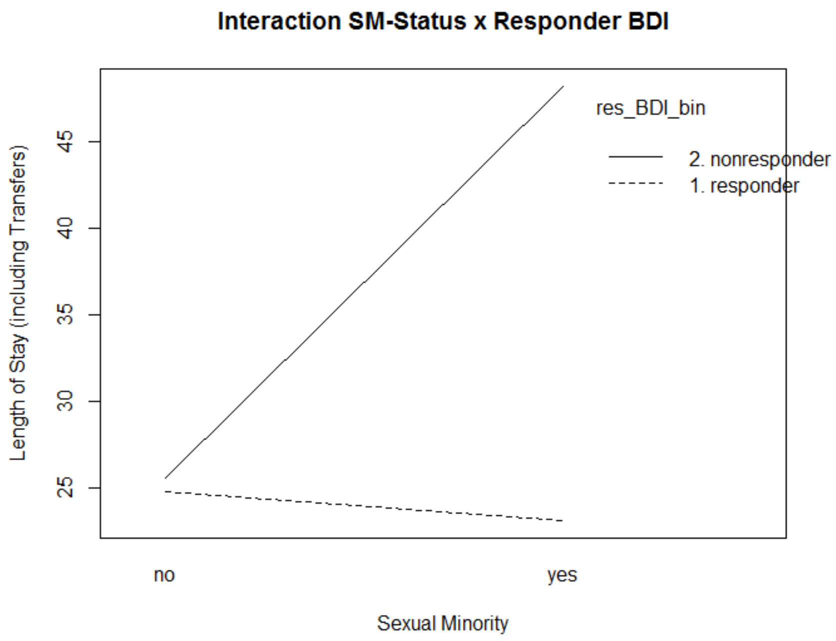

Cohen d responders 0.06 vs. nonresponders 0.73

3.3.2 Adjusting for Confounders

Full model

```
lrm(formula = res_BDI_bin ~ income      + m_languag + F6 + log(length_stay)
*
    sm)
Frequencies of Missing Values Due to Each Variable
  res_BDI_bin income              m_languag           F6     length_stay             sm
           81              0              0            0               0              0


                              Model Likelihood     Discrimination     Rank Discrim.
                                 Ratio Test           Indexes            Indexes
Obs                  552    LR chi2       52.36    R2        0.129    C        0.674
 1. responder        388    d.f.              6    g         0.743    Dxy      0.348
 2. nonresponder 164        Pr(> chi2) <0.0001     gr        2.102    gamma    0.350
max |deriv|        0.02                             gp        0.145    tau-a    0.145
                                                    Brier     0.189
```

```
                     Coef    S.E.   Wald Z Pr(>|Z|)
Intercept           -2.9233 0.7151 -4.09  <0.0001
income              -0.0002 0.0001 -1.84  0.0663
m_languag            1.3052 0.2995  4.36  <0.0001
F6=yes               0.4994 0.2312  2.16  0.0308
length_stay          0.2515 0.1838  1.37  0.1712
sm=yes              -3.7088 1.4048 -2.64  0.0083
length_stay * sm=yes 1.1305 0.4282  2.64  0.0083
```

```
             Effects              Response : res_BDI_bin

 Factor         Low     High    Diff.   Effect    S.E.      Lower 0.95 Upper 0.95
 income      745.39 1537.2 791.82 -0.18265 0.099453 -0.377580  0.012271
  Odds Ratio    745.39 1537.2 791.82  0.83306       NA  0.685520  1.012300
 m_languag      1.00     2.0    1.00  1.30520 0.299500  0.718160  1.892200
  Odds Ratio    1.00     2.0    1.00  3.68830       NA  2.050700  6.633900
 length_stay   14.00    28.0   14.00  0.17435 0.127420 -0.075388  0.424090
  Odds Ratio   14.00    28.0   14.00  1.19050       NA  0.927380  1.528200
 F6 - yes:no    1.00     2.0      NA  0.49936 0.231230  0.046166  0.952550
  Odds Ratio    1.00     2.0      NA  1.64770       NA  1.047200  2.592300
 sm - yes:no    1.00     2.0      NA -0.26707 0.262130 -0.780840  0.246700
  Odds Ratio    1.00     2.0      NA  0.76562       NA  0.458020  1.279800

Adjusted to: length_stay=21 sm=no
```

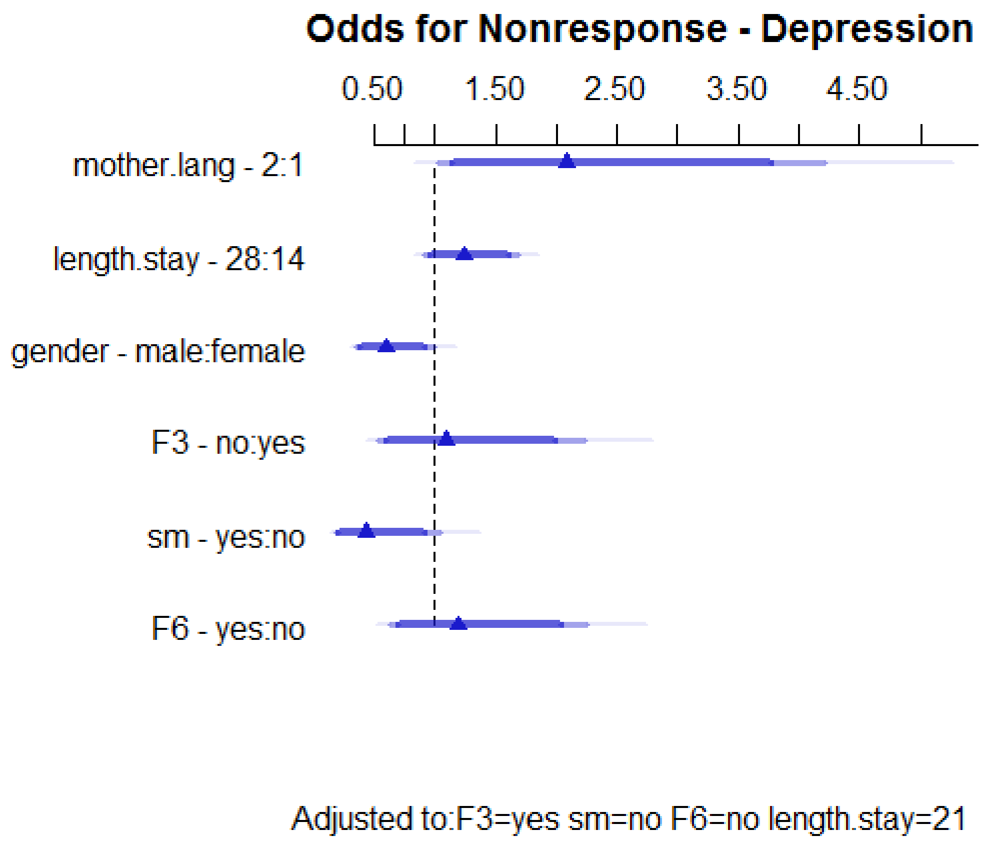

4 Treatment Expectancy

Linear Regression – SM Status as only predictor

```
Residuals:
    Min      1Q  Median      3Q     Max 
-73.127 -15.127   3.873  19.873  26.873 

Coefficients:
            Estimate Std. Error t value Pr(>|t|)    
(Intercept)  73.1275     1.0372  70.503   <2e-16 ***
smyes         0.5748     2.2800   0.252    0.801    
---
Signif. codes:  0 ‘***’ 0.001 ‘**’ 0.01 ‘*’ 0.05 ‘.’ 0.1 ‘ ’ 1

Residual standard error: 23.24 on 631 degrees of freedom
Multiple R-squared:  0.0001007, Adjusted R-squared:  -0.001484 
F-statistic: 0.06356 on 1 and 631 DF,  p-value: 0.801
```

3.4 Interaction with Confounders

3.4.1 Nationality

```
lm(formula = EXPECTANCY ~ sm * nationality)

Residuals:
    Min      1Q  Median      3Q     Max 
-72.778 -14.778   4.017  20.017  34.200 

Coefficients:
                    Estimate Std. Error t value Pr(>|t|)    
(Intercept)           69.857      3.721  18.773   <2e-16 ***
smyes                 16.309      8.265   1.973   0.0489 *  
nationality            2.922      3.192   0.915   0.3604    
smyes:nationality    -14.104      7.122  -1.980   0.0481 *  
---
Signif. codes:  0 ‘***’ 0.001 ‘**’ 0.01 ‘*’ 0.05 ‘.’ 0.1 ‘ ’ 1

Residual standard error: 23.2 on 629 degrees of freedom
Multiple R-squared:  0.006298, Adjusted R-squared:  0.001558 
F-statistic: 1.329 on 3 and 629 DF,  p-value: 0.264
```

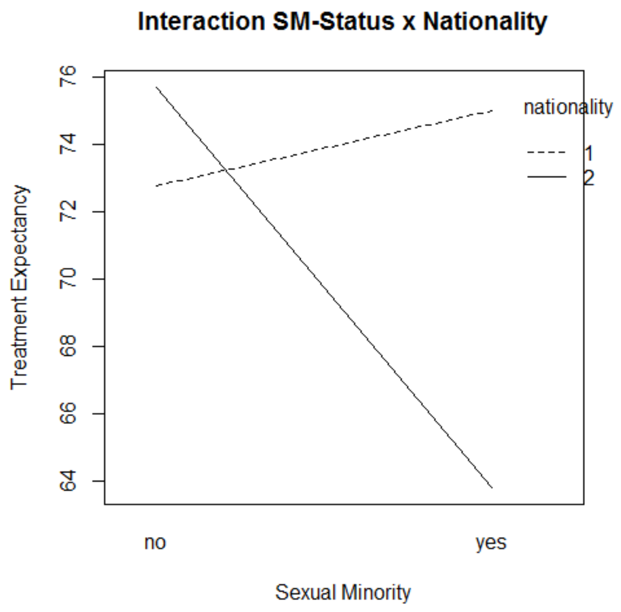

3.5 Adjusting for Confounders

Full Multivariate Model

```
lm(formula = EXPECTANCY ~ age + education + F1 + F3 + F6 + F8 +
    log(length.stay) + nationality * sm)

Residuals:
   Min      1Q  Median      3Q     Max
-76.76 -13.13    3.90   16.91   38.52

Coefficients:
                      Estimate Std. Error t value Pr(>|t|)
(Intercept)           73.33841    6.63933  11.046  < 2e-16 ***
age                    0.33529    0.07592   4.416 1.19e-05 ***
education             -2.71690    0.82170  -3.306 0.000999 ***
F1yes                  3.11889    2.02283   1.542 0.123619
F3yes                 -6.01239    2.17746  -2.761 0.005929 **
F6yes                 -5.46526    2.33113  -2.344 0.019368 *
F8yes                 -7.68336    5.39733  -1.424 0.155079
log(length.stay)      -1.70657    1.46991  -1.161 0.246085
nationality            2.66771    3.10681   0.859 0.390856
smyes                 16.52923    8.05765   2.051 0.040649 *
nationality:smyes    -12.69204    6.97115  -1.821 0.069140 .
---
Signif. codes:  0 '***' 0.001 '**' 0.01 '*' 0.05 '.' 0.1 ' ' 1

Residual standard error: 22.52 on 622 degrees of freedom
Multiple R-squared:  0.07458,  Adjusted R-squared:  0.0597
F-statistic: 5.013 on 10 and 622 DF,  p-value: 4.996e-07
```

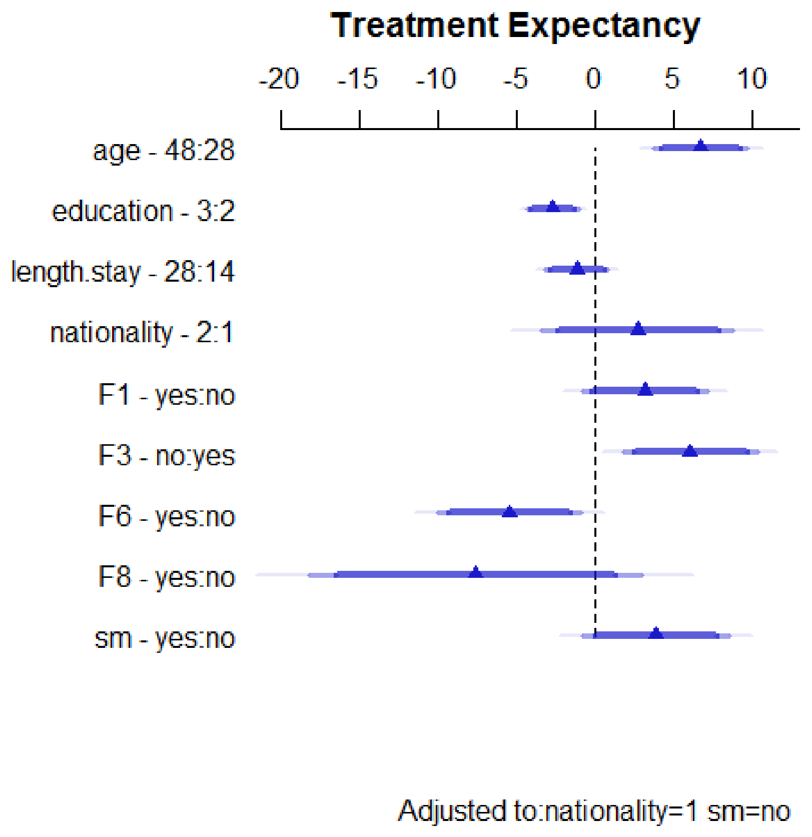

5 Working Alliance

Linear Regression – SM Status as only predictor

```
Residuals:
    Min      1Q  Median      3Q     Max 
-29.542  -5.542   1.458   7.458  12.458 

Coefficients:
            Estimate Std. Error t value Pr(>|t|)    
(Intercept)  47.5420     0.4093 116.140   <2e-16 ***
smyes         0.8301     0.9039   0.918    0.359    
---
Signif. codes:  0 '***' 0.001 '**' 0.01 '*' 0.05 '.' 0.1 ' ' 1

Residual standard error: 9.153 on 627 degrees of freedom
  (4 observations deleted due to missingness)
Multiple R-squared:  0.001343,  Adjusted R-squared:  -0.0002495 
F-statistic: 0.8433 on 1 and 627 DF,  p-value: 0.3588
```

3.6 Interaction with Confounders

Nationality

```
lm(formula = WORKING_ALLIANCE~ sm * nationality)

Residuals:
   Min     1Q Median     3Q    Max 
-29.70  -5.70   1.30   7.30  16.27 

Coefficients:
                    Estimate Std. Error t value Pr(>|t|)    
(Intercept)           49.017      1.460  33.583   <2e-16 ***
smyes                  7.741      3.248   2.384   0.0174 *  
nationality           -1.317      1.252  -1.052   0.2932    
smyes:nationality     -6.196      2.794  -2.218   0.0269 *  
---
Signif. codes:  0 '***' 0.001 '**' 0.01 '*' 0.05 '.' 0.1 ' ' 1

Residual standard error: 9.094 on 625 degrees of freedom
  (4 observations deleted due to missingness)
Multiple R-squared:  0.0173,   Adjusted R-squared:  0.01259 
F-statistic: 3.669 on 3 and 625 DF,  p-value: 0.01217
F-statistic: 1.329 on 3 and 629 DF,  p-value: 0.264
```

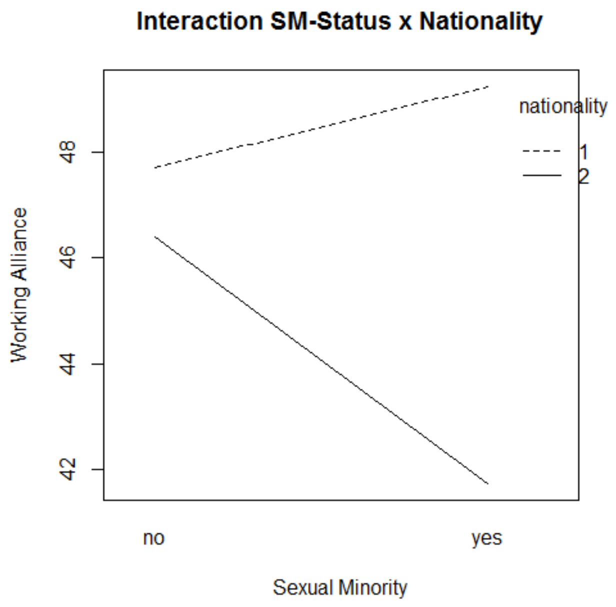

Length of stay

```
lm(formula = WORKING_ALLIANCE~ sm * length_stay)

Residuals:
    Min      1Q  Median      3Q     Max 
-29.545  -5.756   1.175   7.217  14.620 

Coefficients:
                  Estimate Std. Error t value Pr(>|t|)    
(Intercept)       48.18007    0.56586  85.144   <2e-16 ***
smyes              2.91048    1.20213   2.421   0.0158 *  
length_stay       -0.02648    0.01643  -1.612   0.1075    
smyes:length_stay-0.06416    0.02856  -2.246   0.0250 *  
---
Signif. codes:  0 '***' 0.001 '**' 0.01 '*' 0.05 '.' 0.1 ' ' 1

Residual standard error: 9.041 on 625 degrees of freedom
  (4 observations deleted due to missingness)
Multiple R-squared:  0.02876,	Adjusted R-squared:  0.0241 
F-statistic: 6.169 on 3 and 625 DF,  p-value: 0.0003902
```

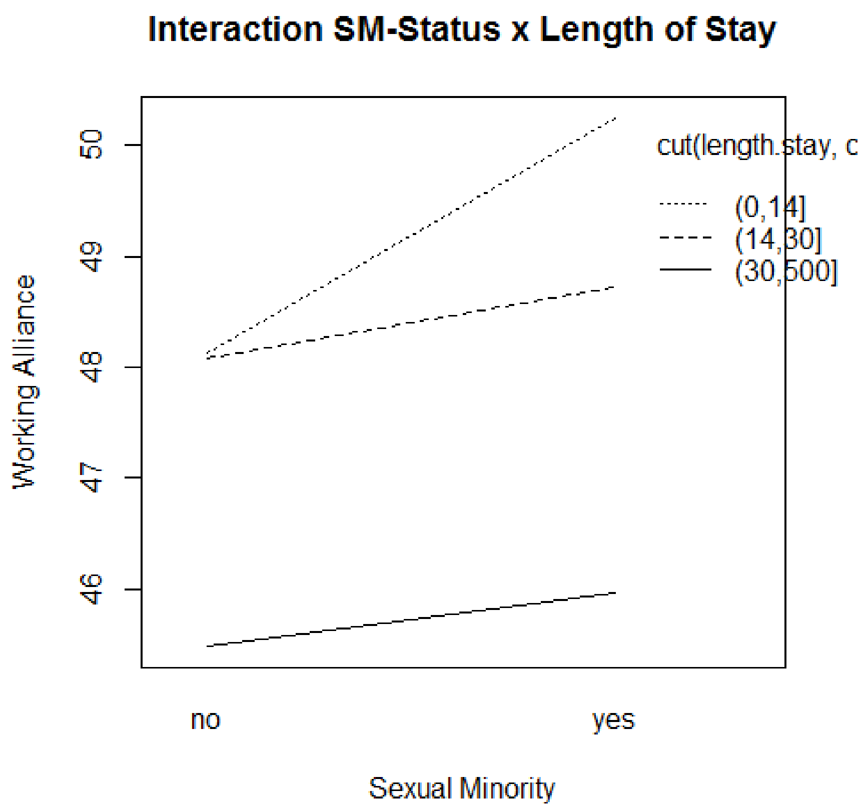

3.7 Adjusting for Confounders

Full Multivariate Model

```
lm(formula = WORKING_ALLIANCE~ age + income + mother.lang + F6 + 
nationality * 
    sm + log(length.stay) * sm)

Residuals:
    Min      1Q  Median      3Q     Max 
-27.846  -5.006   1.324   6.783  15.705 

Coefficients:
                         Estimate Std. Error t value Pr(>|t|)    
(Intercept)            53.1870257  2.8033334  18.973  < 2e-16 ***
age                     0.0372529  0.0300199   1.241 0.215098    
income                  0.0008752  0.0003190   2.744 0.006251 ** 
mother.lang            -2.1360386  1.3435372  -1.590 0.112377    
F6yes                  -3.2776815  0.9042098  -3.625 0.000313 ***
nationality            -0.4411467  1.3462041  -0.328 0.743252    
smyes                  12.7364565  5.0569053   2.519 0.012032 *  
log(length.stay)       -1.6213674  0.6572585  -2.467 0.013900 *  
nationality:smyes      -5.1899957  2.7390247  -1.895 0.058580 .  
smyes:log(length.stay) -1.6774997  1.3338478  -1.258 0.208996    
---
Signif. codes:  0 ‘***’ 0.001 ‘**’ 0.01 ‘*’ 0.05 ‘.’ 0.1 ‘ ’ 1

Residual standard error: 8.796 on 619 degrees of freedom
  (4 observations deleted due to missingness)
Multiple R-squared:  0.08961,  Adjusted R-squared:  0.07637 
F-statistic:  6.77 on 9 and 619 DF,  p-value: 2.678e-09
```

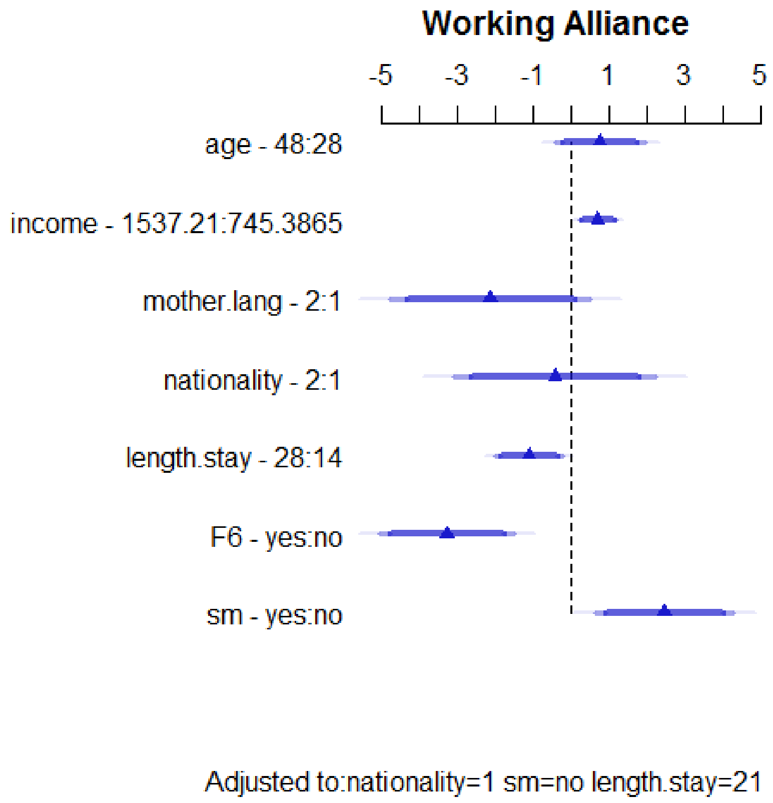

Supplement: Additional file 1: — Supplement-additional-results-statistical-analysis-in-paper. (PDF 687 kb) [file 12888_2017_1337_MOESM1_ESM.pdf]
